# Morphology of *Stephanella hina* (Bryozoa, Phylactolaemata): common phylactolaemate and unexpected, unique characters

**DOI:** 10.1186/s40851-020-00165-5

**Published:** 2020-11-10

**Authors:** Thomas F. Schwaha, Masato Hirose

**Affiliations:** 1 Department of Evolutionary Biology, University of Vienna, Althanstraße 14, 1090 Vienna, Austria; 2 Kitasato University, School of Marine Biosciences, Kitasato 1-15-1, Sagamihara-Minami, Kanagawa 252-0373 Japan

**Keywords:** Stephanellidae, Bryozoan evolution, Epistome, Myoanatomy

## Abstract

*Stephanella hina* is a little studied freshwater bryozoan belonging to Phylactolaemata. It is currently the only representative of the family Stephanellidae, which in most reconstructions is early branching, sometimes even sister group to the remaining phylactolaemate families. The morphological and histological details of this species are entirely unknown. Consequently, the main aim of this study was to conduct a detailed morphological analysis of *S. hina* using histological serial sections, 3D reconstruction, immunocytochemical staining and confocal laser scanning microscopy techniques. The general morphology is reminiscent of other phylactolaemates; however, there are several, probably apomorphic, details characteristic of *S. hina*. The most evident difference lies in the lophophoral base, where the ganglionic horns/extensions do not follow the traverse of the lophophoral arms but bend medially inwards towards the mouth opening. Likewise, the paired forked canal does not fuse medially in the lophophoral concavity as found in all other phylactolaemates. Additional smaller differences are also found in the neuro-muscular system: the rooting of the tentacle muscle is less complex than in other phylactolaemates, the funiculus lacks longitudinal muscles, the caecum has smooth muscle fibres, latero-abfrontal tentacle nerves are not detected and the medio-frontal nerves mostly emerge directly from the circum-oral nerve ring. In the apertural area, several neurite bundles extend into the vestibular wall and probably innervate neurosecretory cells surrounding the orifice. These morphological characteristics support the distinct placement of this species in a separate family. Whether these characteristics are apomorphic or possibly shared with other phylactolaemates will require the study of the early branching Lophopodidae, which remains one of the least studied taxa to date.

## Introduction

Phylactolaemate bryozoans are, from an evolutionary perspective, very interesting, as they comprise the sister group to the two remaining taxa, Stenolaemata and Gymnolaemata [1, 2]. They are a small group of ~ 80 recent species, and due to lack of calcification and hard tissues, they rarely fossilize. Several characteristics are typical of this taxon: a horseshoe-shaped lophophore (suspension-feeding tentacle crown), body-wall musculature and an epistome—a flap-like ciliated bulge protruding over the mouth opening, probably involved in feeding [3, 4]. Morphological analyses usually unite bryozoans with brachiopods and phoronids as “lophophorates” due to similarities in their general body plan, including an epistome-like structure in all groups and, at least in some representatives of the other two taxa, a horseshoe-shaped lophophore (e.g., [5, 6]). This concept initially gained almost no support through molecular studies but has recently been supported in more recent phylogenies (e.g., [7]). Nonetheless, the phylactolaemate body plan is, from a morphological perspective, the most similar to other lophophorate taxa. Hence, their study is not only important for gaining more insight into the ground pattern of bryozoans and their character evolution but also for making possible phylogenetic inferences on a broad scale.

Six to seven families are currently distinguished among Phylactolaemata, with the large bulk of species belonging to the Plumatellidae, and all other families consisting of one to five species. Several older studies agreed that the genus *Stephanella*, the sole genus of the Stephanellidae, represents an early branch within phylactolaemate diversification, a notion also supported by more recent molecular analyses [8, 9]. Most studies concerning this genus dealt with its taxonomy and distribution [10, 11]. In addition to the original description from Oka [12], one study has applied more sophisticated methods to investigate the morphology of this species [13]. Only recently, *Stephanella hina* was documented and analysed properly for the first time [14]. Several peculiarities, such as the very thin connections between individual zooids within a colony and their tube-like cystids, are unique to phylactolaemates. In addition, the body wall secretes a mostly unconnected tube rather than a cuticular ectocyst, as found in other bryozoans, which is also unique for this species [14]. In the past decade, fluorescence staining methods and confocal laser scanning microscopy have been increasingly applied to the study of adult bryozoans [e.g., 15–21]. Since *S. hina* remains an important representative, the aim of this work was to use histological as well as fluorescence techniques to investigate this neglected species in more detail to compare it with other phylactolaemates.

## Material & Methods

Samples were collected from 2014 to 2016 in Tsukuba or a nearby pond as previously described by Schwaha et al. [14]. Samples were either fixed for sectioning in 2–2.5% glutaraldehyde in 0.01 M sodium cacodylate buffer (pH 7.4) for approximately 2 h or for fluorescence staining in 4% paraformaldehyde in 0.1 M phosphate buffer (pH 7.4) for 1 hour. Samples were afterwards rinsed several times in the corresponding buffer and further processed or stored in buffer containing ~ 0.1% NaN3 until further preparation. Further processing for sectioning and fluorescence staining was conducted as previously described (see, e.g., [15, 16]).

## Results

### General morphology

**Fig. 1:**
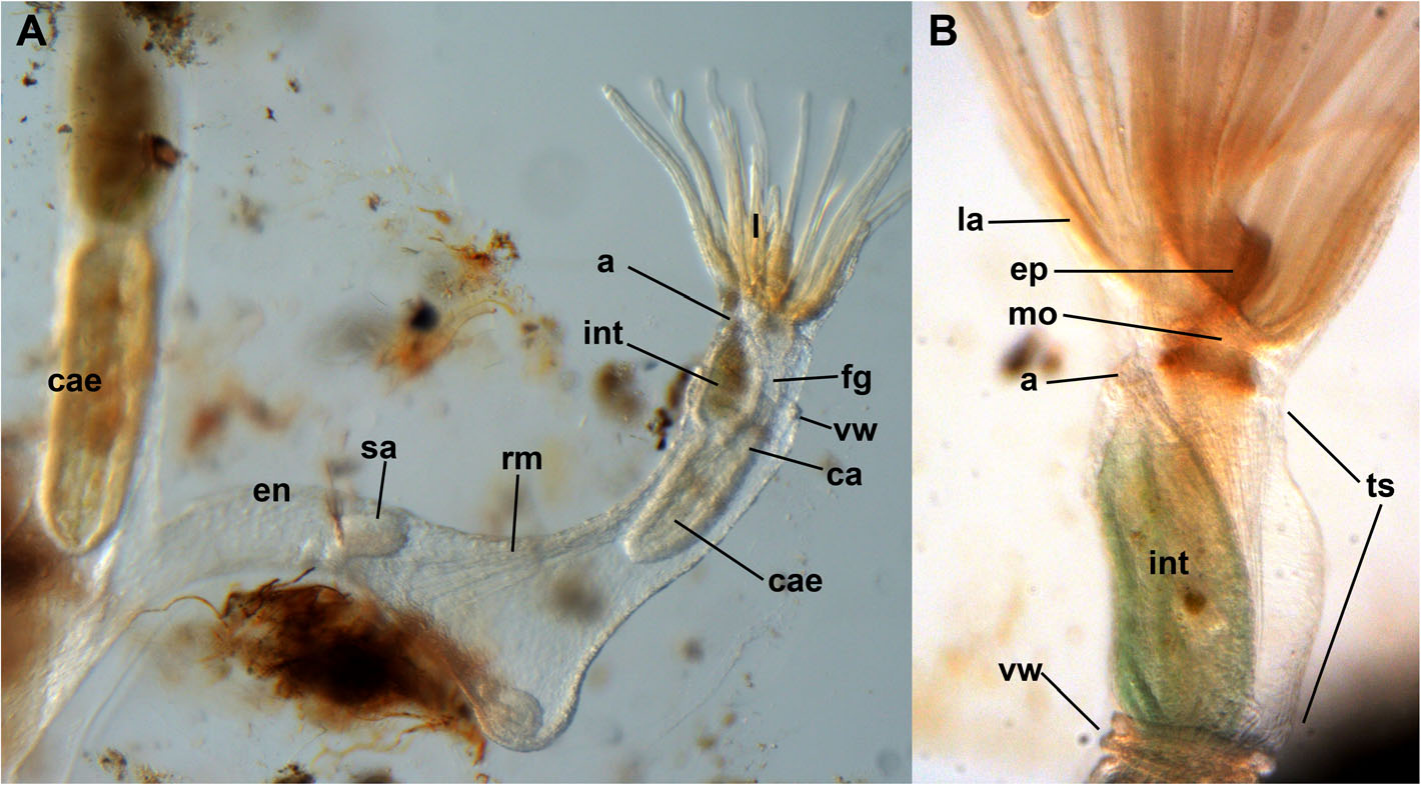
General overview of *Stephanella hina*. Live specimens. **a** Lateral view of two zooids on the budding tip, with a younger zooid on the right and an older zooid on the left. **b** Detail of a single zooid including several morphological characteristics. Abbreviations: a - anus, ca - cardia, cae - caecum, en - endocyst, ep - epistome, fg - foregut, int - intestine, l - lophophore, la – lophophoral arm, mo - mouth opening, rm. - retractor muscle, sa - statoblast anlage, ts - tentacle sheath, vw - vestibular wall

**Fig. 2:**
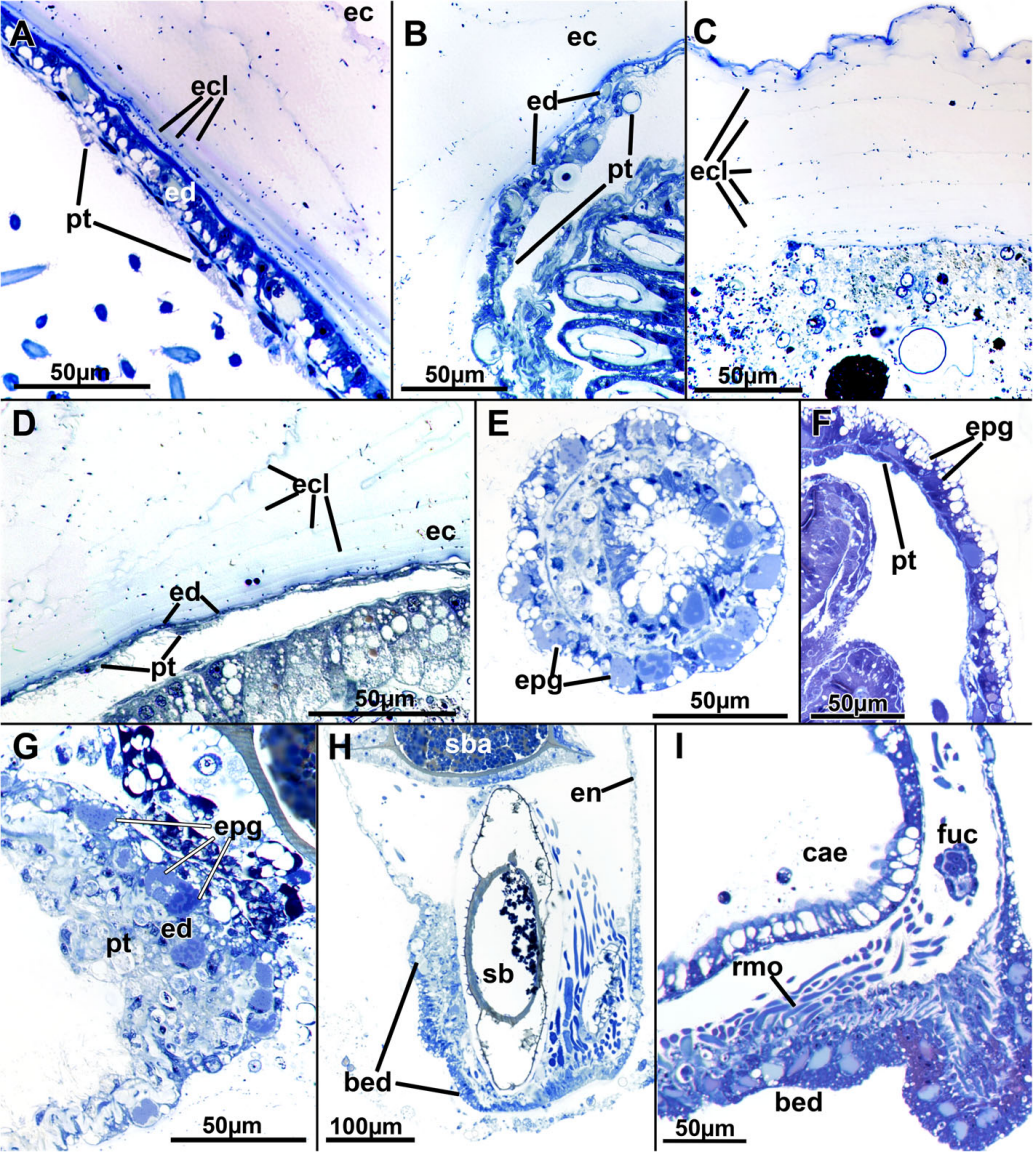
Histological details of the cystid wall of *Stephanella hina*. Semithin sections. **a-d** The ectocyst secretion consists of various concentric layers as seen by their granular stained borders. **a** Close to the endocyst, several distinct thin layers are discernible. The epidermis is prominent, while the peritoneum is not. **b** Body wall with thin epidermis and thicker, prominent peritoneum. **c** Distinct layers of the ectocyst are visible. The ectocyst stains only weakly, but its uppermost layer (top of image) is often more conspicuous. **d** Endocyst with extremely thin epidermis and peritoneum. **e-g** Epidermal glandular parts of the body wall. **e** Section of numerous translucent vesicles and other more distinctly stained vesicles. **f** Similar to E, longitudinal section. **g** Endocyst with a prominent peritoneal layer, probably containing lytic tissues and substance. **h** Longitudinal section of a zooid showing prominent glandular epidermis at the basal side (bottom of image) where the animal is attached to the substrate and the anchorage of the prominent retractor muscle fibres is located. **i** Detail of the basal epidermal attachment pad showing a high abundance of glandular vesicles. Abbreviations: bed - basal epidermis, cae - caecum, ec - ectocyst, ecl - ectocyst layers, en - endocyst, ed. - epidermis, epg - epidermal glands, fuc - funiculus, pt. - peritoneum, rmo - retractor muscle origin, sb - statoblast, sba - statoblast anlage

**Fig. 3:**
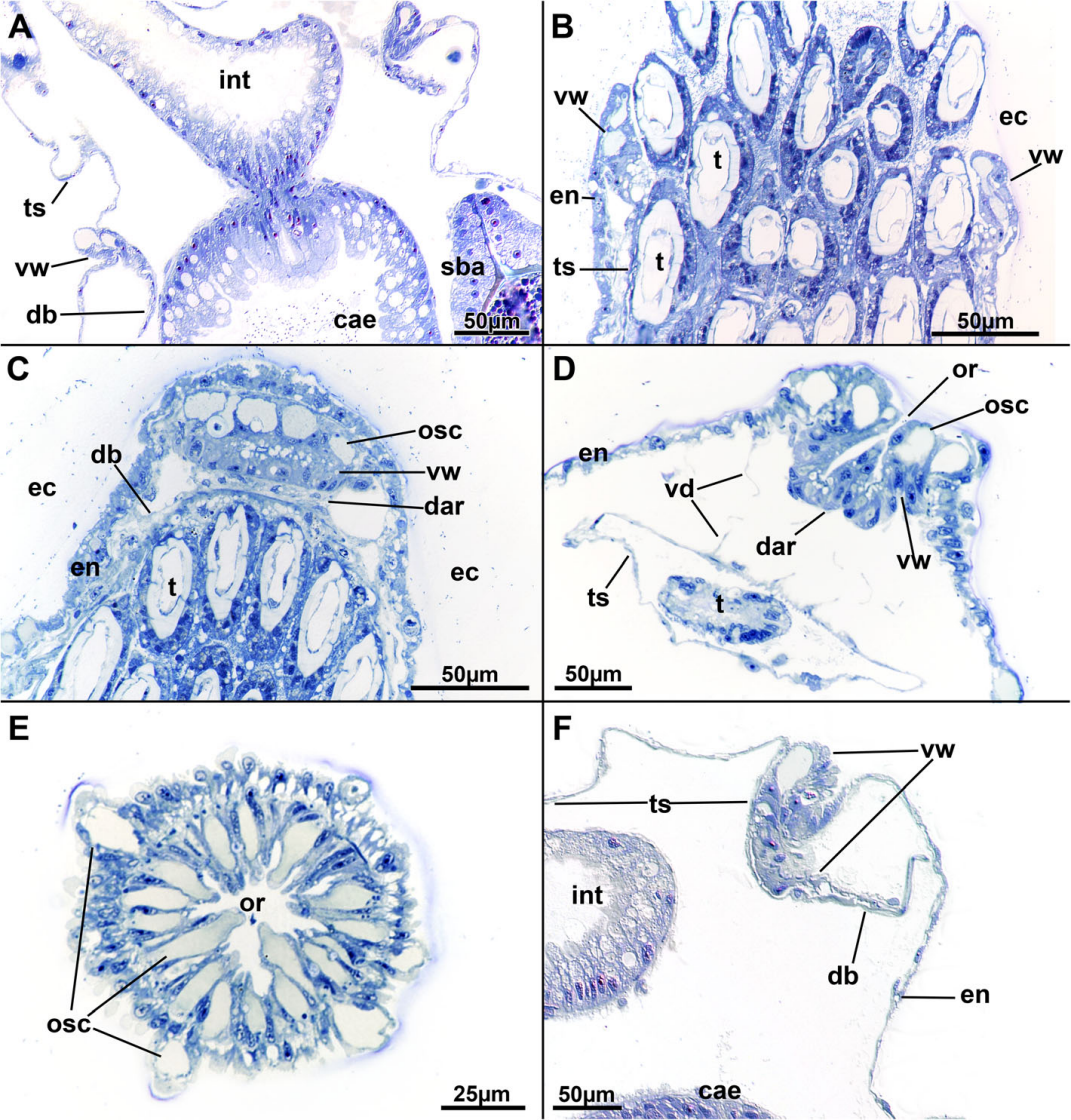
Histological details of the vestibular area of *Stephanella hina*. Semithin sections. **a** Longitudinal section of an extended polypide. **b** Oblique section of a slightly extended specimen showing the apertural area. **c** Longitudinal section through the vestibular wall of a zooid with retracted polypide showing the dense glandular/sensory (?) orificial cells. **d** Median longitudinal section of the apertural area of a zooid with retracted polypide. **e** Cross-section of the folded vestibular wall with contracted vestibular wall musculature. **f** Close-up of the vestibular wall of a protruded specimen (compare to A). Abbreviations: cae - caecum, dar - diaphragmatic area, db - duplicature band, ec - ectocyst, en - endocyst, int - intestine, or - orifice, osc – orificial (sensory?) cells, sba - statoblast anlage, t - tentacle, ts - tentacle sheath, vd - vestibular dilatators, vw - vestibular wall

**Fig. 4:**
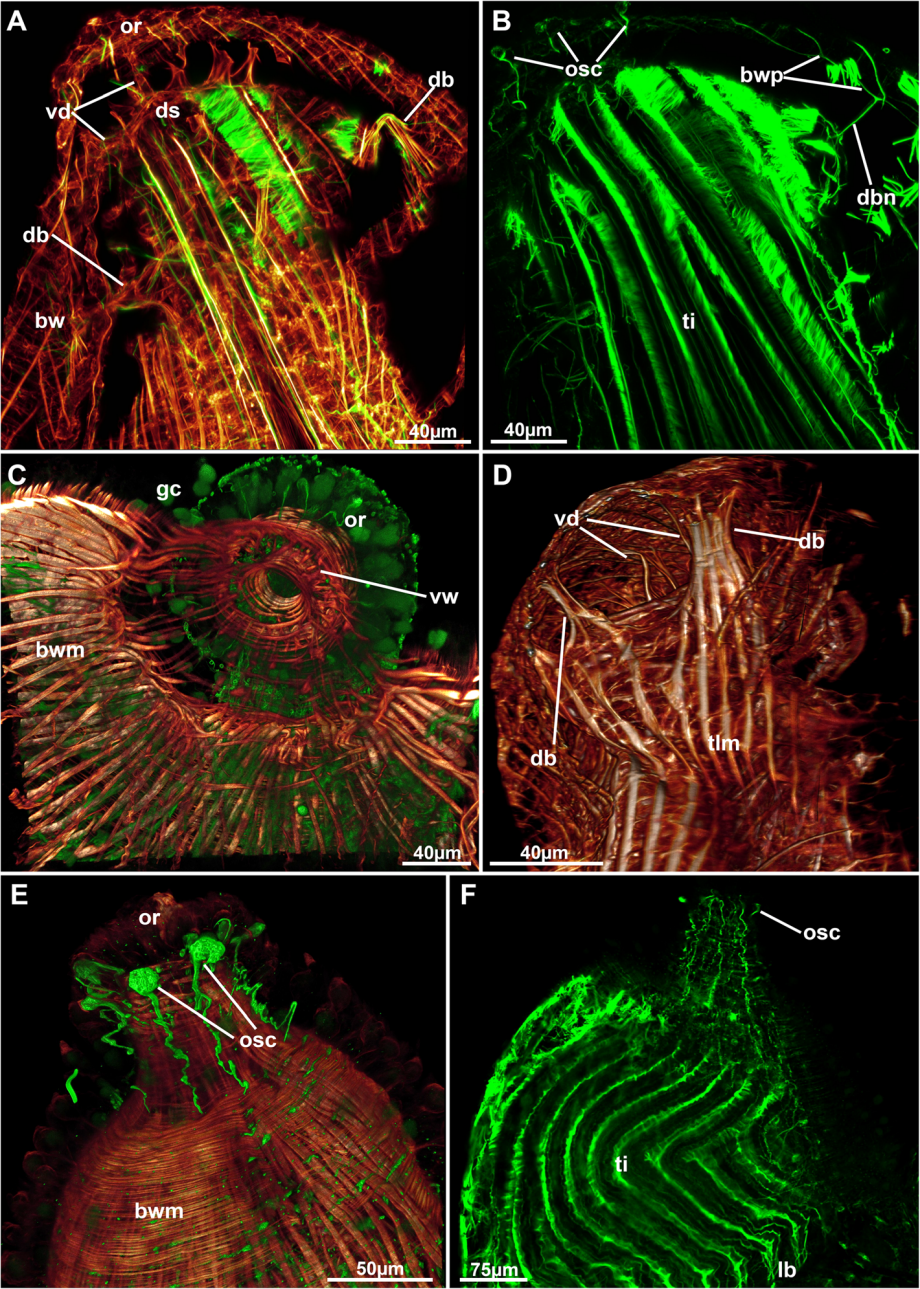
Neuro-muscular system of the apertural area of *Stephanella hina*. Confocal laser scanning microscopy stacks based on acetylated alpha-tubulin labelling. Volume renderings, projections or optical slices. Acetylated alpha-tubulin in green LUT, f-actin in glow LUT. **a** Projection of a zooid with a retracted polypide showing duplicature bands and vestibular dilatators. **b** Thin neurite bundles traversing the duplicature bands and extending towards the conspicuous orificial cells. **c** Volume rendering of the orificial area of a zooid with a retracted polypide showing a closed vestibular area surrounded by stained gland cells. **d** Lateral view of a retracted zooid showing duplicature bands and vestibular dilatators. **e** Details of specific orificial cells. **f** Thin neurite bundles extending along the distal body wall towards the orificial cells. Abbreviations: bw – body wall, bwm – body wall musculature, bwp – body wall nervous plexus, db – duplicature band, dbn – duplicature band neurites, ds – diaphragmatic sphincter, gc – gland cell, or – orifice, osc – orificial (sensory?) cells, ti – tentacle innervation, tlm – tentacle sheath longitudinal muscles, vd – vestibular dilatators, vw – vestibular wall

The zooids of a colony have an attached basal part and an attached vertical tube that contains the polypide extending from it. The attached basal part shows elongated extensions that interconnect zooids in a colony (see [14] and Fig. 1a). The cystid wall consists of the cellular endocyst and extracellular ectocyst (Fig. 2). At the distal tip of the cystid lies the orifice (Fig. 3d, e), where polypides protrude from or retract into. At the margin of the cystid, the vestibular wall folds proximally (Fig. 3). It is short but always of prominent thickness (Figs. 1; 3). The cells at its distal border always show a large vacuole of homogenous content (Fig. 3), which at least partially also stain for anti-acetylated tubulin (Fig. 4b, e). Proximally, the vestibular wall terminates at the diaphragm (Fig. 3d), which characteristically possesses a distinct sphincter muscle [17]. The sphincter is not clearly distinguishable from the remaining circular muscles of the vestibular wall in *Stephanella hina*. From the diaphragm, the thin introvertable tentacle sheath extends towards the lophophoral base (Fig. 1b). The horseshoe-shaped lophophore carries approximately 32–34 ciliated tentacles and surrounds the central mouth opening. From the anal side, a ciliated flap, the epistome, protrudes slightly above the mouth opening (Fig. 1b). The mouth opening enters the u-shaped gut, which terminates via the anus in the tentacle sheath.

### Cystid structure

The body wall is bi-layered and consists of an outer epidermis and an inner peritoneal layer. For the most part, the endocyst is thin, but locally thicker areas of the epidermal layer are present. These areas are glandular and are frequently encountered in the epidermal layer (Fig. 2e–i), although distinct cellular inclusions can also sometimes be found in the peritoneum (Fig. 2b). Overall, both layers of the endocyst can have increased thickness, and variants with one layer being thicker than the other or both being of equal thickness have been encountered (Fig. 2). Distinct reasons for this variation could not be found but might be linked with the ectocyst thickness (see below). Only at the basal part, where the zooid is directly attached to the substrate and from where the prominent retractor muscles originate, can a prominent endocyst always be ascertained (Fig. 2h, i). Glandular cells in the endocyst appear mostly vacuolar with a spacious cavity of homogenous or heterogeneous content (Fig. 2e–g, i). In addition, a second type of gland cell with numerous, smaller translucent cavitations or vesicles occurs in the epidermis.

External to the epidermis lies the ectocyst, which has only little contact with the epidermis and is secreted as multilayered tubes (see also Schwaha et al. 2016). During histological preparation, it shrinks extensively and covers the epidermis more closely. The ectocyst is a translucent, sticky hull secreted by the endocyst. It consists of numerous concentric layers that on sections are discernible as concentric rings containing densely stained, minute particles. The outer- and innermost layers often stain more intensely and conspicuously than the middle layers (Fig. 2a, c, d). Close to the epidermis, these layers appear denser, whereas the outer margin shows larger gaps between each layer (Fig. 2a, c).

### Lophophore

**Fig. 5:**
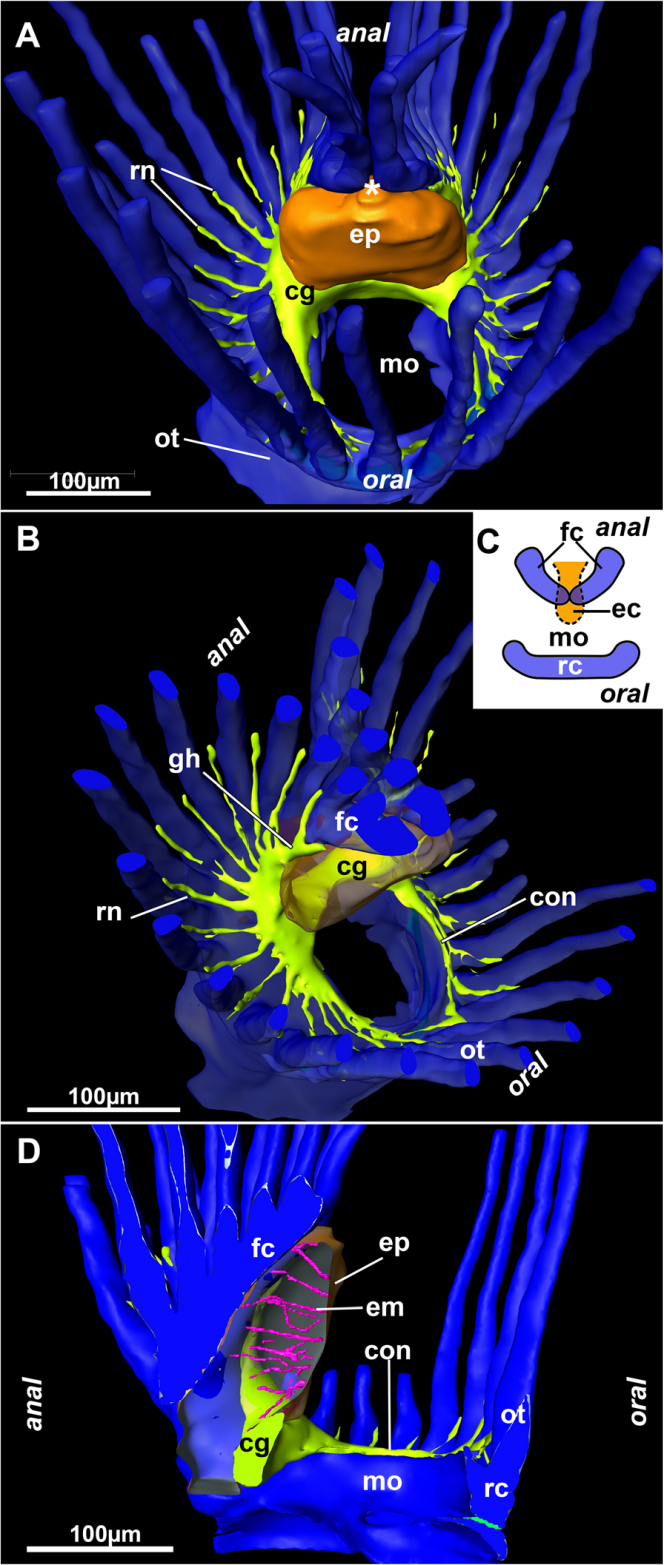
3D reconstruction of serial semithin sections of the lophophoral base of *Stephanella hina*. Nervous system in yellow, epistome in orange and coelomic cavity in blue*.* **a** Oral view of the lophophoral base showing the epistome above the cerebral ganglion. Note that the inner lophophoral arc (forked canals) is not fused (asterisk). **b** Oblique view of the lophophoral base with the epistome displayed transparently. **c** Schematic drawing of the three coelomic canals, the forked canal and the epistome canal on the anal side and the ring canal on the oral side. **d** Lateral view of the epistome including the muscles traversing its cavity. Abbreviations: cg - cerebral ganglion, con - circumoral nerve ring, ec – epistomial coelom, em – epistome muscles, ep - epistome, fc – forked canal, gh - ganglionic horn, mo - mouth opening, ot - oral tentacles, rc – ring canal, rn - radial nerve

**Fig. 6:**
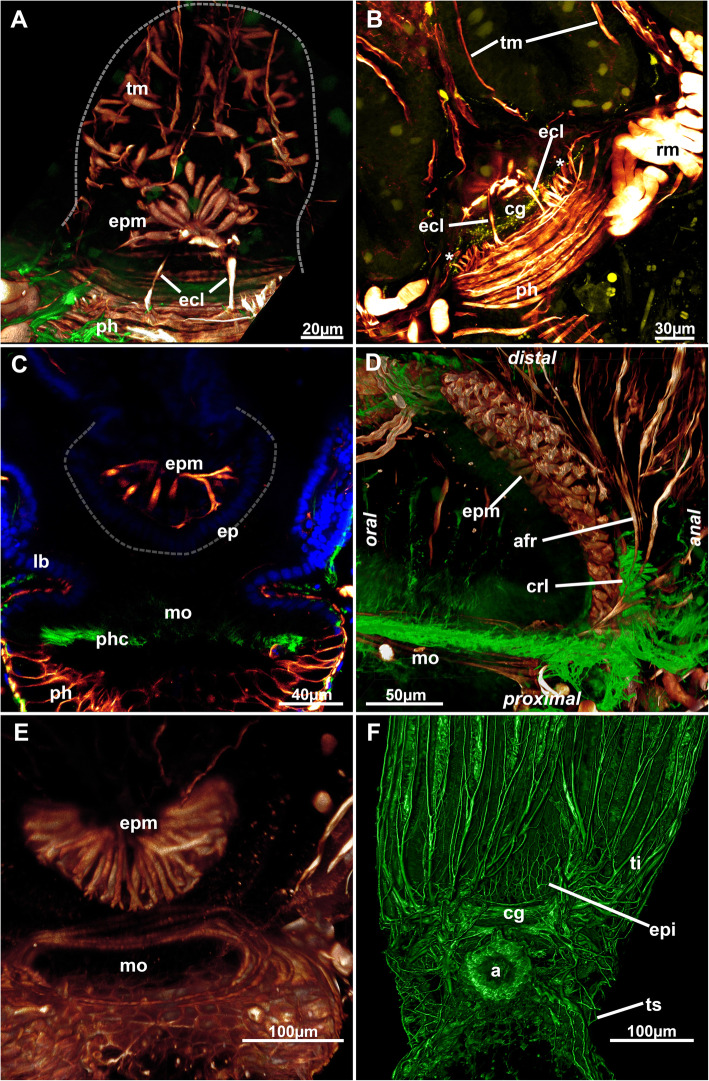
Epistome myoanatomy and innervation of *Stephanella hina*. Confocal laser scanning microscopy stacks based on acetylated alpha-tubulin labelling. Volume renderings, projections or optical slices. Acetylated alpha-tubulin in green LUT, f-actin in glow LUT. **a** Volume rendering of the epistomial musculature from the anal side. Note the lateral epistomial canal muscles. **b** Maximum projection of the same volume in A showing thin lateral muscle fibres lateral to the epistomial canal muscles. **c** Optical section through the foregut and the epistome showing individual, smooth muscle fibres crossing. **d** Lateral rendering showing epistomial musculature and dense lateral ciliation on their proximo-lateral side. **e** Volume rendering of the epistomial musculature. **f** Innervation of the epistome. A thin plexus extends directly from the cerebral ganglion. Abbreviations: a – anus, afr – abfrontal muscle root, cg – cerebral ganglion, crl – ciliary ridge laterally of the epistome, ecl – epistome canal muscles, ep – epistome, epi – epistome innervation, epm – epistome musculature, lb. – lophophoral base, mo – mouth opening, ph – pharynx, phc – pharynx ciliation, rm. – retractor muscle, ti – tentacle innervation, tm – tentacle muscles, ts – tentacle sheath

The tentacle crown, or lophophore, of *Stephanella hina* is horseshoe shaped and has rather short lophophoral arms (Figs. 1; 5). On the oral side of the lophophore, there are eight tentacles supplied by the ring canal situated at the lophophoral base and showing lateral open connections to the remaining body cavity. The lateral tentacles and most on the lophophoral arms are largely confluent with the remaining coelomic cavity. Only the tentacles on the anal side above the epistome show two additional canalized extensions (Fig. 5). The openings of these canals are wide and located at the lophophoral base, approximately in the area of the cerebral ganglion. The canals show dense ciliation of the inner peritoneal lining (Fig. 6d), and each supply two canals that protrude above the epistome coelom medially but do not fuse in their median plane (Fig. 5). The epistome coelom is an extension from the remaining body cavity between the oral and anal gut shanks reaching above the cerebral ganglion, which is located on the anal side of the pharynx, and protrudes medially in the direction of the mouth opening.

**Fig. 7:**
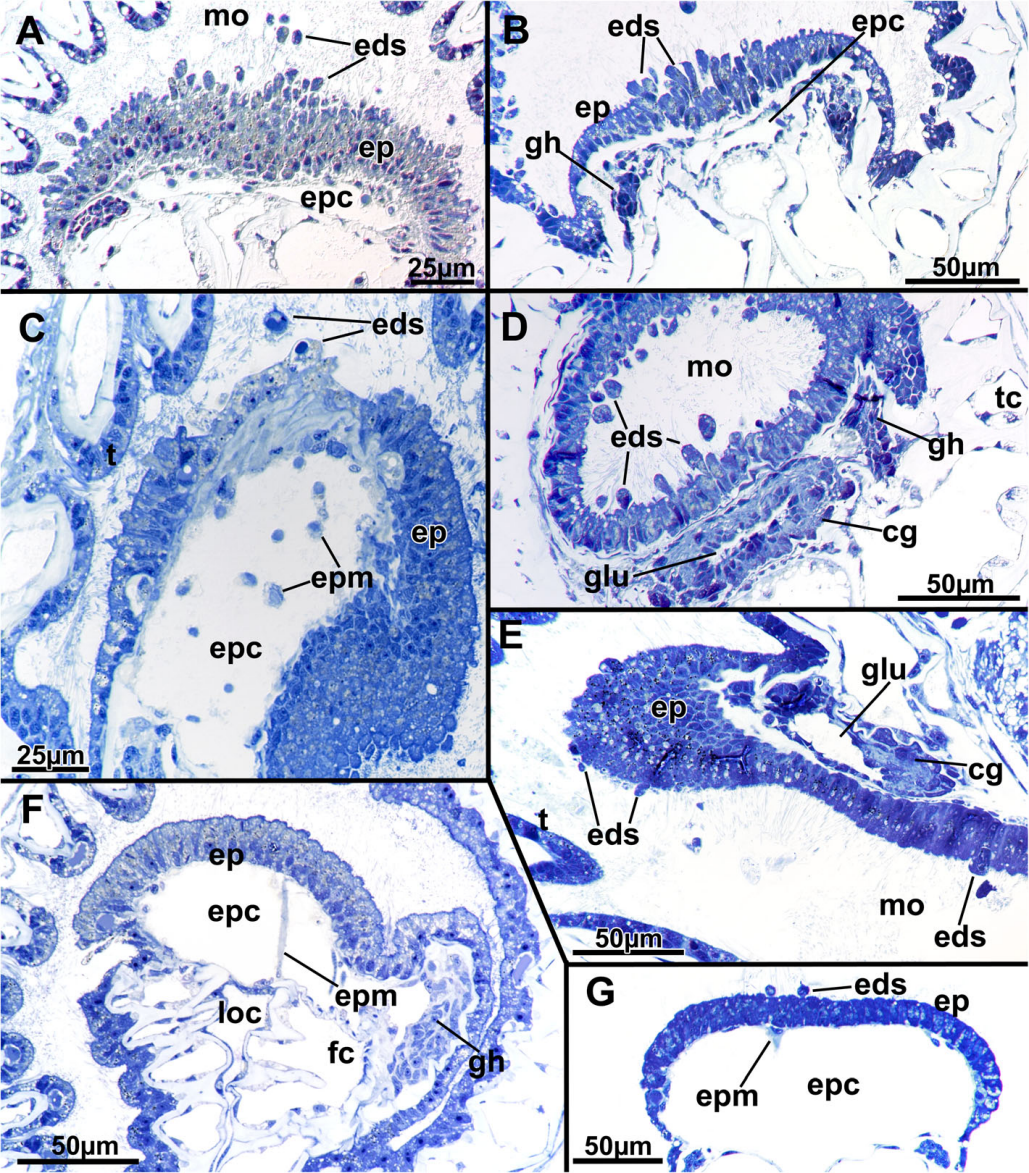
Histological details of the epistome area of *Stephanella hina*. Semithin sections. All images are cross-sections (or slightly oblique) except E, which is a longitudinal image. With the exception of the latter, the epistomial epidermis always faces up towards the oral side of the animal and the anal side facing down. **a** The epistome epidermis is prominent and shows distinct epidermal secretions that ultimately enter the mouth area in the lophophoral base. **b** Less prominent epistome epidermis with fewer epidermal secretions. **c** An epistome epidermis with the median part apparently forming secretion droplets. Below that area, a conspicuous extracellular matrix seems to be present. **d** Section through the cerebral ganglion slightly below the epistome showing similar epidermal secretions occurring in the epidermal lining of the gut. **e** Longitudinal sections showing few secretion events in the epistome but synchronously in the foregut epithelium slightly below the cerebral ganglion. **f** An epistome showing prominent cells containing multiple inclusions but lacking any epidermal secretions. **g** Inconspicuous epistome with few, small secretions. Abbreviations: cg - cerebral ganglion, eds - epidermal secretions, ep - epistome, epc - epistome coelom, epm - epistome musculature, gh - ganglionic horn, glu - ganglion lumen, loc - lophophoral concavity, mo - mouth opening, t - tentacle, tc - tentacle coelom

The epidermal lining of the epistome shows great variation in its histological differentiation. It always consists of highly prismatic elongated cells stretching from the lophophoral base towards the mouth opening (Fig. 7). The cells of the epistome epithelium can be inconspicuous (Fig. 7g) or only show numerous (partially osmiophilic) secretory droplets or vesicles (Fig. 7f), but in the majority of observations, it shows active secretion of globular vesicles in a holocrine manner (Fig. 7a-c, e, g). Such secretions are encountered in various stages from bulges from the epistome epidermis (Fig. 7e) to elongated, stalked cells with only little contact with the remaining epithelium (Fig. 7b) to separate globules pinched off from the epithelium or within the area of the mouth opening (Fig. 7a-c, g). A similar condition is present at the beginning of the digestive tract, in the epithelium proximal to the epistome (Fig. 7d). These secretory activities are reflected by the reddish-brownish coloration of these areas in live animals (Fig. 1).

**Fig. 8:**
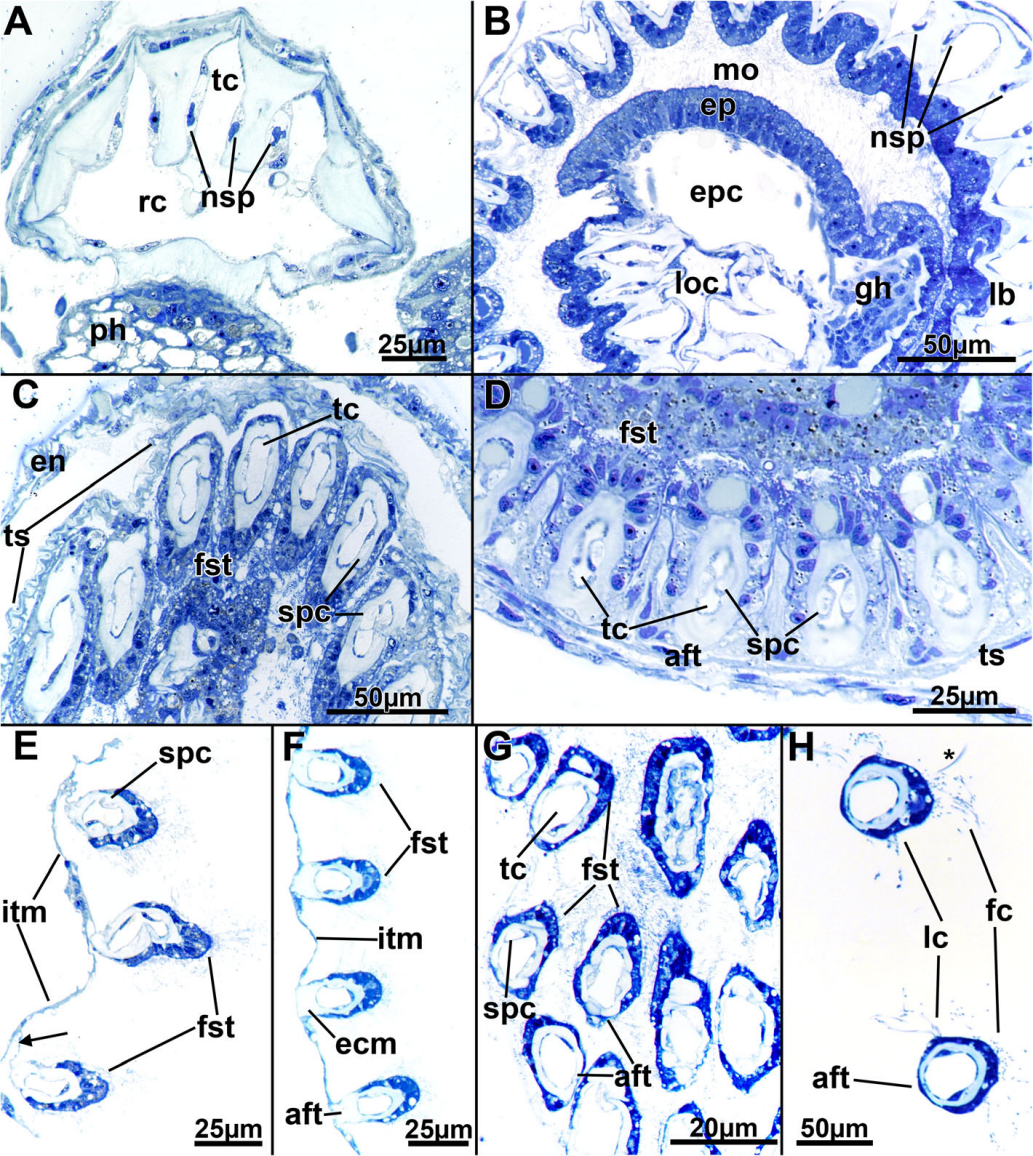
Histological details of the tentacles of *Stephanella hina*. Semithin sections. **a** Longitudinal section of the oral lophophoral base through the ring canal showing extension from the ring canal into the oral tentacles and longitudinally sectioned nuclei of the subperitoneal cells of the tentacles. **b** Slightly oblique cross-section of the lophophoral base. The tentacles on the right side are more proximal, with the tentacle coelom still embedded in a common extracellular matrix, whereas the sectioned tentacles on the left show individual tentacles already. The nuclei of the subperitoneal cells are visible on the right side. **c** Cross-section of retracted tentacles within the tentacle sheath. Note also the subperitoneal cells medially occluding the tentacle coelom in some areas of the retracted positions (right side of the image). **d** Close-up of cross-sectioned retracted tentacles showing subperitoneal cells bulging medially towards the tentacle coelom in the longitudinal axis of each tentacle. **e** & **f** Cross-sections of extended lophophores showing different attachment possibilities of the tentacles to the intertentacular membrane via thin pedicels (**e**) or with the abfrontal side of the tentacles completely integrated into the membrane (**f**). (**g** & **h**) Cross-sections of densely arranged tentacles (**g**) with a thin epidermal layer always present on the abfrontal side and a thicker epidermal layer on the frontal side. Cilia are mainly located on the lateral and frontal sides of the tentacles (**h**). Note a thick cilium (asterisk) extending from the upper tentacle in (**h**), possibly a latero-frontal cilium. Abbreviations: aft - abfrontal side of tentacle, ecm - extracellular matrix, en - endocyst, ep - epistome, epc - epistome coelom, fc - frontal cilia, fst - frontal side of tentacle, gh - ganglionic horn, itm - intertentacular membrane, lb. - lophophoral base, lc - lateral cilia, loc - lophophoral concavity, mo - mouth opening, ph - pharynx, nsp - nuclei of subperitoneal cells, rc - ring canal, spc - subperitoneal cells of tentacles, tc - tentacle coelom, ts - tentacle sheath

**Fig. 9:**
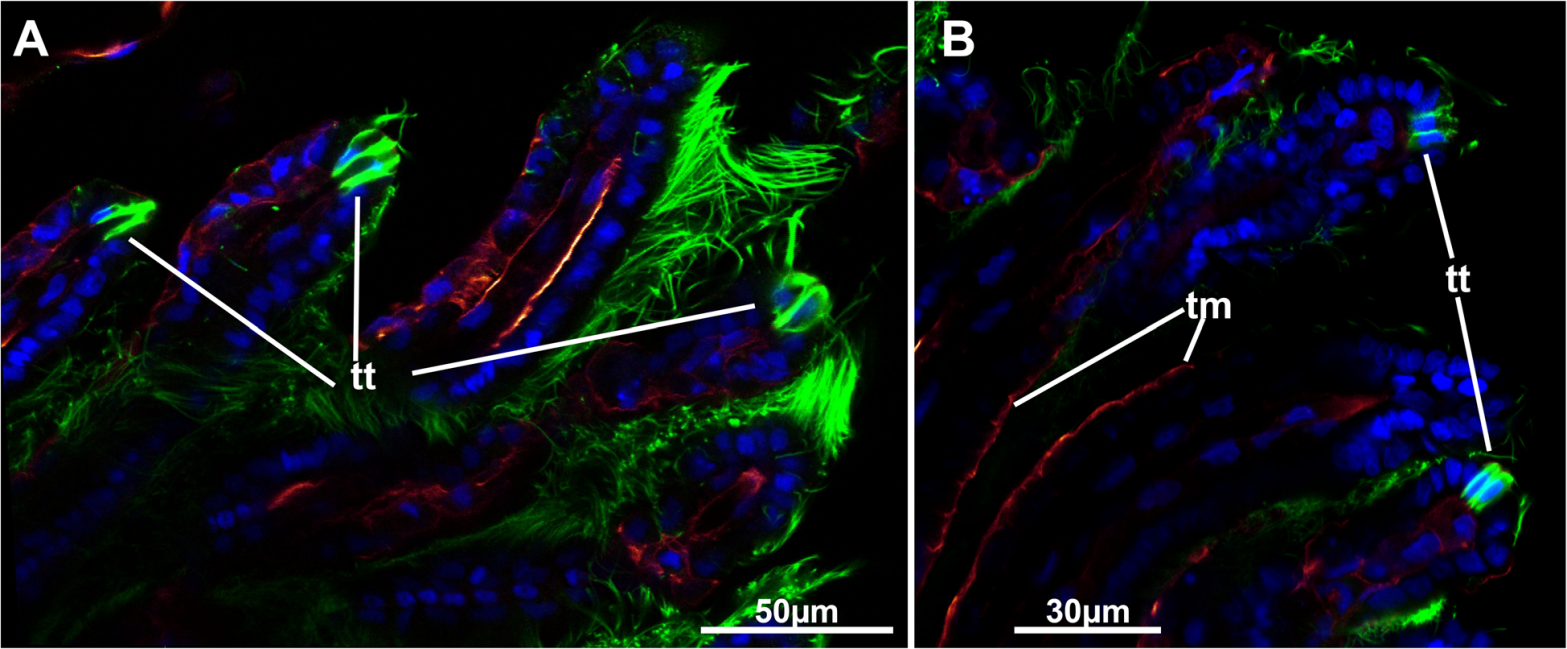
Tentacle tips of *Stephanella hina*. Confocal laser scanning microscopy stacks based on acetylated alpha-tubulin labelling. Optical slices. Acetylated alpha-tubulin in green LUT, f-actin in glow LUT. **a** & **b** Different sections of tentacle tips showing distinct ciliary tips at the distal ends of the tentacles. Abbreviations: tm – tentacle muscle, tt – tentacle tip

The tentacles are proximally united at the lophophoral base. In cross-section, the tentacles typically show a frontal side facing the mouth opening and an abfrontal side facing the opposite direction. Frontally, the epidermal lining is thickest and gradually diminishes in size from the lateral towards the abfrontal side, where the epithelium is only thin (Fig. 8c-h). Frontal and lateral cilia are readily distinguishable in the tentacles (Fig. 8h), whereas distinct latero-frontal cilia are not detected at this level of resolution. Medially, a considerable extracellular matrix separates the outer epidermal layer from the inner peritoneal lining of each tentacle coelom (Fig. 8). The latter is always thin and bordered on each lateral side by a pair of subperitoneal cells. On sections, these appear homogenously filled (Fig. 8c, d, g). In retracted polypides, the subperitoneal cells often locally bulge medially and suppress the tentacle coelom to the frontal and abfrontal sides (Fig. 8c, d). These cells appear to accompany each tentacle over its entire length, and the nuclei of these cells are only encountered directly at the lophophoral base, shortly before the tentacles individualize from the remaining lophophoral base (Fig. 8a, b). At the tip of each tentacle, a distinct ciliary bundle is present. It seems to be restricted to a few cells in the epidermal tip (Fig. 9).

A distinct intertentacular membrane interconnects each tentacle on their abfrontal sides, close to the lophophoral base. It is merely a thin epidermal duplicature that either is directly integrated into the epithelium of the abfrontal side of each tentacle (Fig. 8f) or has a thin pedicle extending from the membrane to the abfrontal side of tentacles (Fig. 8e).

### Digestive tract and funiculus

**Fig. 10:**
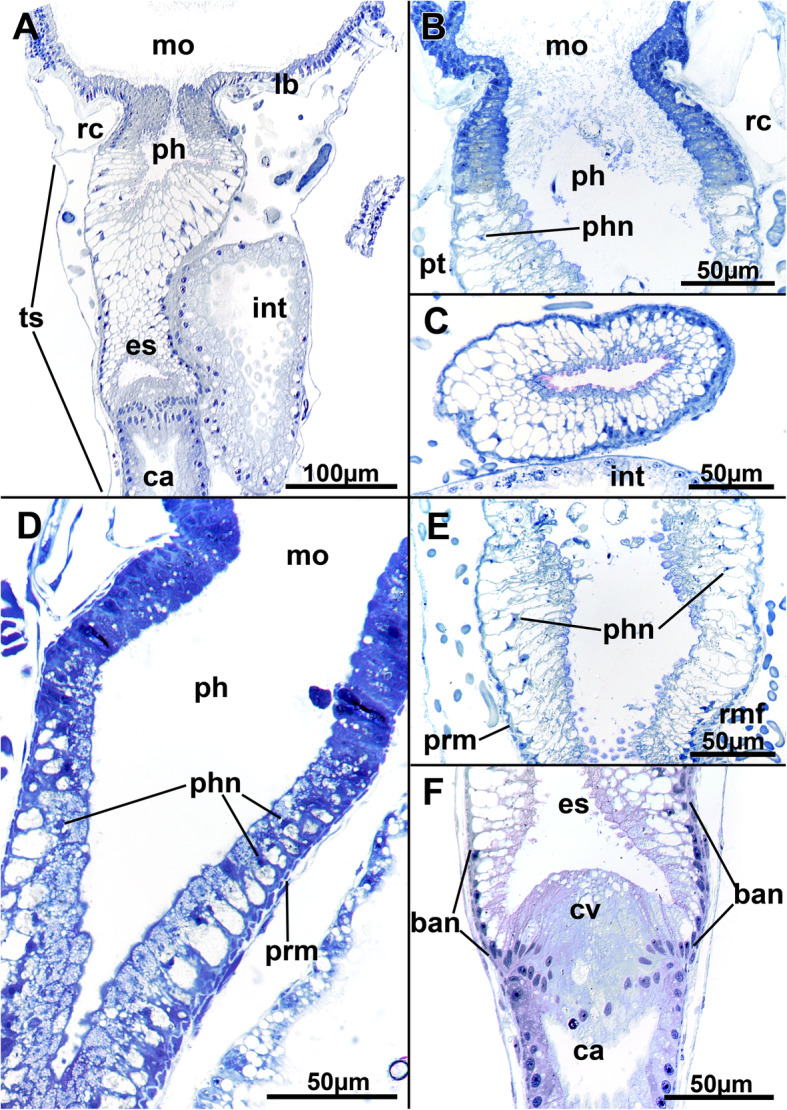
Histological details of the foregut of *Stephanella hina*. **a** Longitudinal section of an extended polypide showing the mouth opening, foregut until the cardia, and intestine. **b** Detail of a mouth opening and pharynx area in a zooid with retracted polypide. The nuclei of the pharynx appear pycnotic and are wedged between large vacuoles in the cells of the epithelium. **c** Cross-section of a pharynx showing the highly vacuolar appearance of its epithelium. **d** Longitudinal section of the mouth opening and pharyngeal area showing a high abundance of smaller vesicles in the apical part of the pharyngeal epithelial cells. **e** Detail of the lower part of the pharynx entering the esophagus. **f** The esophageal-cardia boundary is marked by the cardiac valve. On the esophagus, basally located nuclei are present in its cells. Abbreviations: ban - basal nuclei, ca - cardia, cv - cardiac valve, es - esophagus, int - intestine, lb. - lophophoral base, mo - mouth opening, ph - pharynx, phn - nucleus of the pharynx, prm - pharyngeal ring muscles, rc - ring canal, rmf - retractor muscle fibres, ts - tentacle sheath

**Fig. 11:**
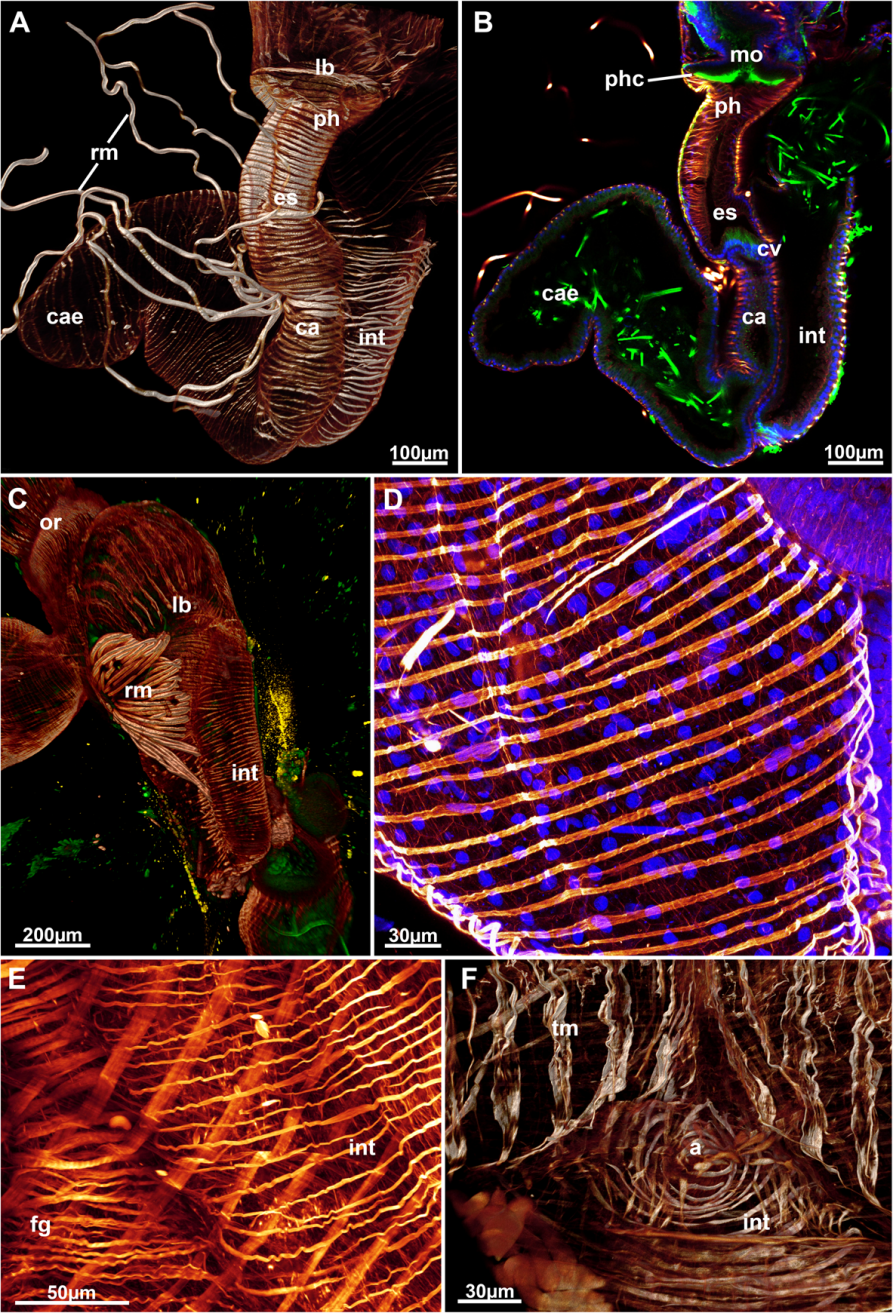
Muscular system of the digestive tract of *Stephanella hina*. Confocal laser scanning microscopy stacks based on f-actin labelling. Volume renderings, projections or optical slices. F-actin in glow LUT. **a** Volume rendering of a dissected zooid showing the major areas of the gut. **b** Optical section of the data set in A showing the border between the esophagus and the cardia. **c** Volume rendering showing the fanned retractor muscle inserting into various sites of the oral digestive tract. **d** Detail of the smooth circular muscles of the caecum. **e** Detail of the circular muscles of the foregut showing cross-striation and the smooth hindgut, the intestine. **f** View of the circular muscles of the anus. Abbreviations: a – anus, ca – cardia, cae – caecum, cv – cardiac valve, es – esophagus, fg – foregut, int – intestine, lb. – lophophoral base, mo – mouth opening, or – orifice, ph – pharynx, phc – pharynx ciliation, rm. – retractor muscle, tm – tentacle muscles

**Fig. 12:**
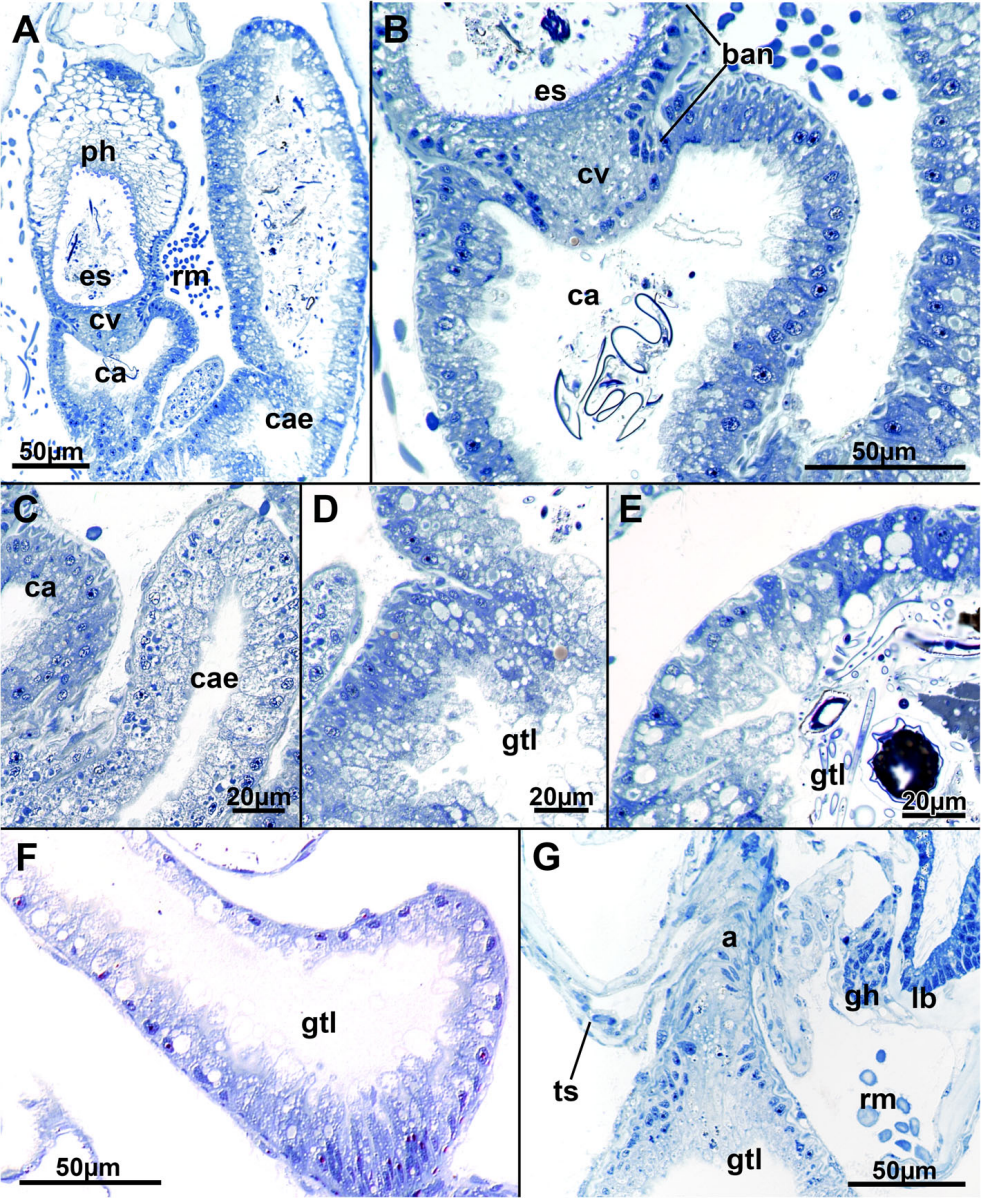
Histological details of the mid- and hindgut of *Stephanella hina*. **a** Longitudinal section of a polypide showing the transition of the esophagus to the cardia and parts of the caecum. **b** Close-up of the cardiac valve and cells of the cardia. **c**–**e** Detailed images of the cellular architecture of the caecum epithelium showing its variable cellular architecture. **f** Details of the cells of the intestine. **g** Detail of the anal area entering the tentacle sheath. Abbreviations: a - anus, ban - basal nuclei, ca - cardia, cae - caecum, cv - cardiac valve, es - esophagus, gh - ganglion horn, gtl - gut lumen, lb. - lophophoral base, ph - pharynx, rm. - retractor muscles, ts – tentacle sheath

The digestive tract of *Stephanella hina* consists of three distinct regions: fore-, mid- and hindgut (Fig. 1). At the lophophoral base, the mouth opening continues into the pharynx followed by the esophagus, which both represent the foregut. The area of the mouth opening has highly prismatic cells with distinct ciliation (Fig. 10a, b, d) that often forms a flat ciliated disc (in protruded polypides) (Figs. 10a; 11b). Proximal to the ciliated area, the pharynx is characterized by a prominently vacuolated epithelium, which shows some variation among analysed specimens (Fig. 10a–e). Apically towards the gut lumen, several smaller vesicular inclusions can be found to various extents (Fig. 10b, d, e). The remaining epithelium is filled with spacious vacuoles (Fig. 10). The nuclei of the pharyngeal epithelium are located in the middle of the baso-apical axis and appear pycnotic (Fig. 10a–e). The adjacent esophageal area shows similar cytological details to the upper pharynx but is much thinner in its extent and has basally located nuclei in the epithelium (Fig. 10f). The midgut consists of a tubular cardia separated from the esophagus by the cardiac valve (Figs. 10f; 11a, b; 12a, b). Cardiac epithelial cells show apical extensions that appear to be secretions released into the gut lumen (Fig. 12b). Proximally, the cardia enters the voluminous caecum, which in *S. hina* shows a high disparity regarding its histological structure. Its cells show various degrees of cellular inclusions (Fig. 12c–e). Some areas of the caecum show distinct, intensely stained areas in its lining (Fig. 12e). The hindgut or intestine is the last distinct part of the gut and continues on the anal side of the caecum. It forms a small ellipsoid chamber that enters the tentacle sheath with the anus approximately at the height of the lophophoral base and mouth opening (Figs. 1; 12g). The cells of the intestine are not as prominent as the midgut and show only small vesicular inclusions (Fig. 12f).

**Fig. 13:**
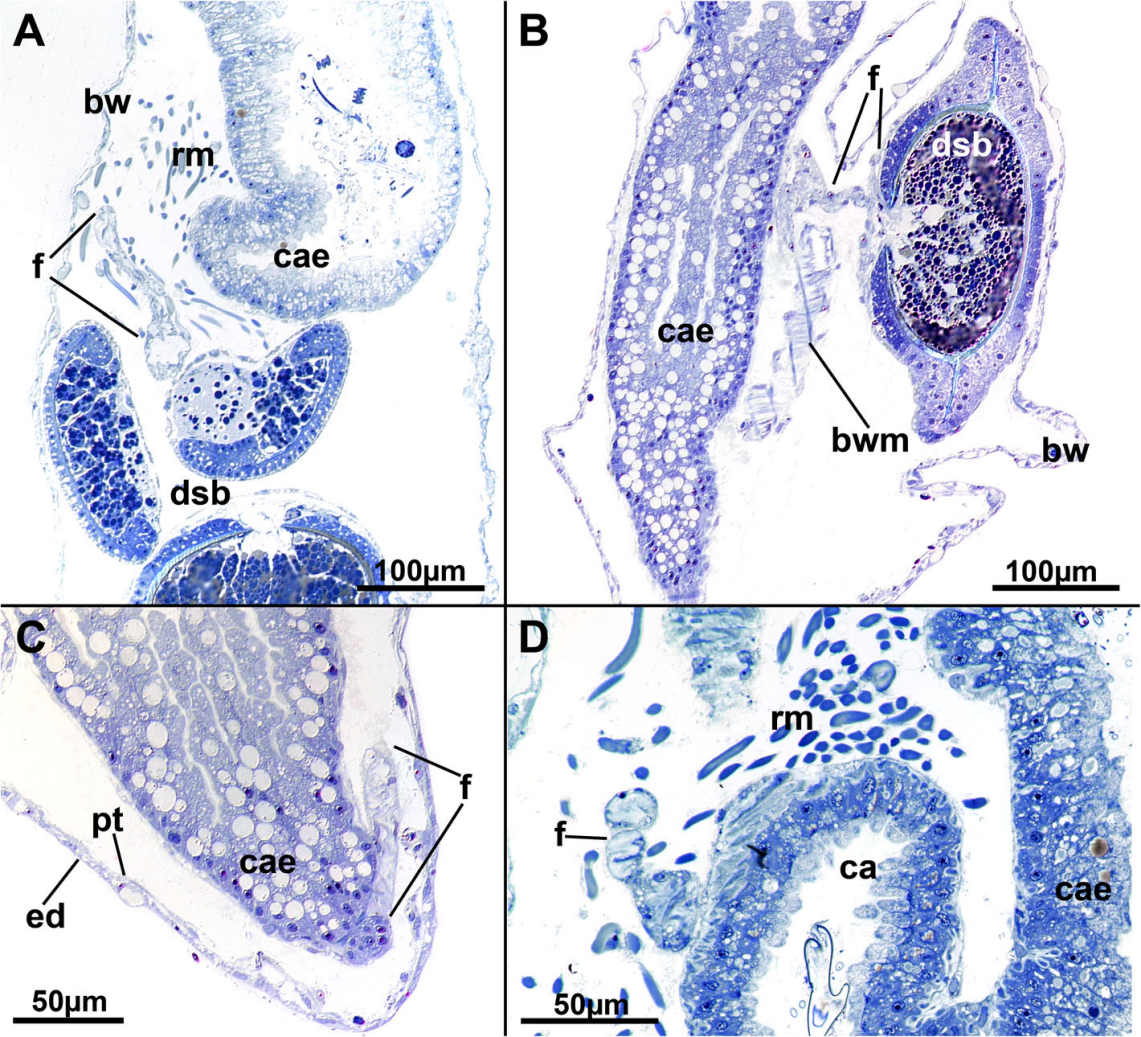
Histological details of the funiculus of *Stephanella hina*. All longitudinal sections. **a** Funiculus in close proximity to the caecum and several developing statoblasts, which are also covered by a thin lining of funicular epithelium. **b** A funicular strand that encloses a very progressed statoblast anlage. The funicular lining surrounding the latter is very thin. **c** Proximal end of the caecum showing the origin of the funiculus. **d** Close-up of a funicular strand, which consists of a thin epithelium surrounding a small central lumen. Abbreviations: bw - body wall, bwm - body wall musculature, ca - cardia, cae - caecum, dsb - developing statoblast, ed. - epidermis, f - funiculus, pt. - peritoneum, rm. - retractor muscles

At the proximal end of the caecum, a thin peritoneal cord, the funiculus, extends as an elongated tube towards the basal body wall (Fig. 13c). Its lining cells are mostly thin, and in retracted polypides, the funiculus forms several loops within the body cavity (Fig. 13). Statoblasts are formed in the funiculus and in several specimens can be found at several locations. The funicular epithelium forms a regular, thin wrapping around each statoblast anlage (Fig. 13a, b).

### Muscular system

**Fig. 14:**
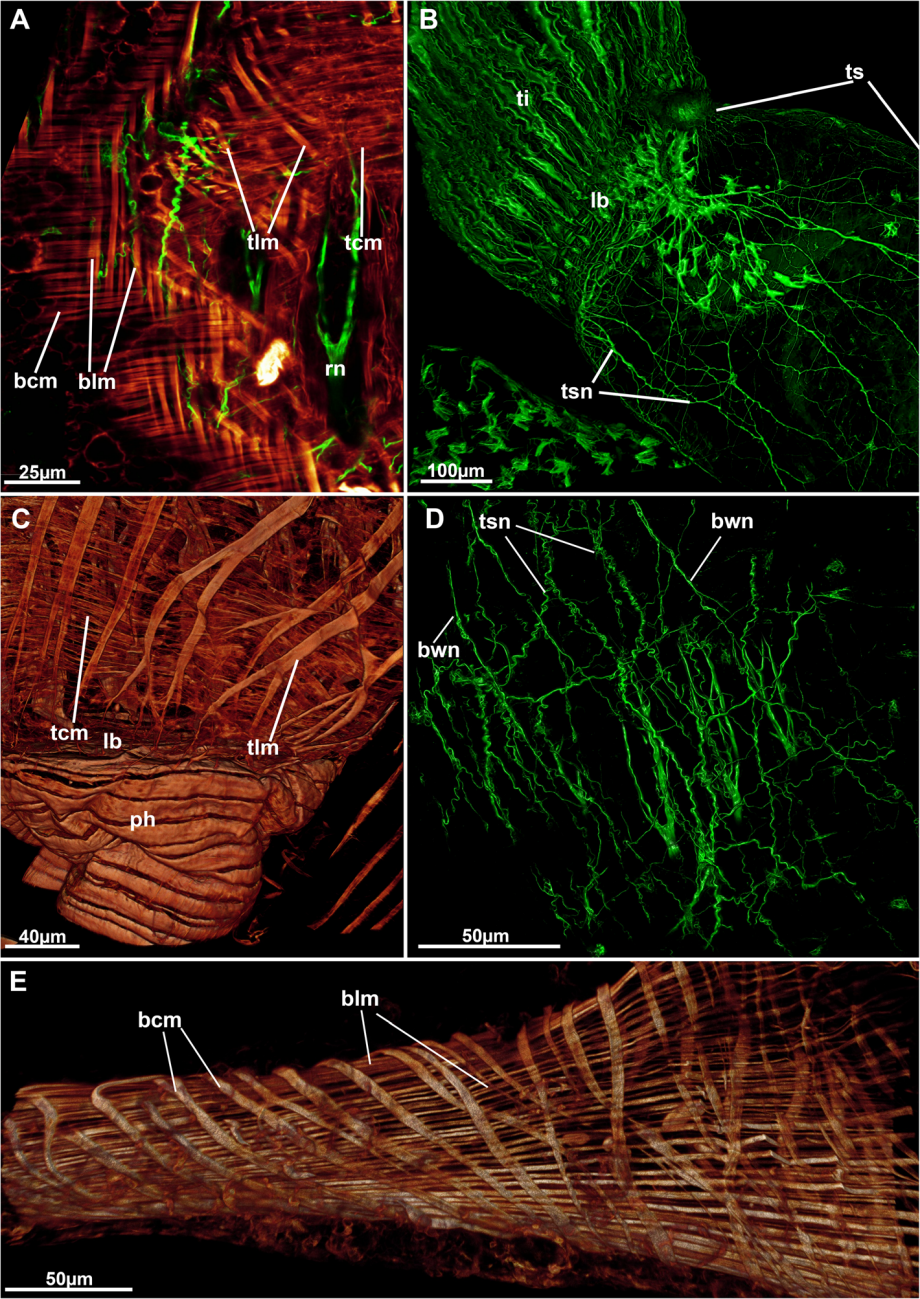
Neuro-muscular system of the peripheral areas of *Stephanella hina*. Confocal laser scanning microscopy stacks based on acetylated alpha-tubulin labelling. Volume renderings, projections or optical slices. Acetylated alpha-tubulin in green LUT, f-actin in glow LUT. **a** Optical section showing body wall musculature on the left and tentacle sheath musculature on the right. Both show thick longitudinal fibres and thinner circular fibres. **b** Overview of an extended polypide showing diffuse tentacle sheath innervation in form of a plexus. **c** Tentacle sheath musculature of a retracted polypide showing both circular and longitudinal fibres close to the lophophoral base. **d** Diffuse nerve plexus of the body wall and partially underlying tentacle sheath nervous plexus. **e** Orthogonal grid of the body wall musculature in the thin, ‘stolon’-like parts of the colony. Abbreviations: bcm – body wall circular muscle, blm – body wall longitudinal muscle, bwn - body wall nerves, lb. – lophophoral base, ph – pharynx, rn – radial nerve, tcm – tentacle sheath circular muscle, ti – tentacle innervation, tlm – tentacle sheath longitudinal muscle, ts – tentacle sheath, tsn – tentacle sheath innervation

**Fig. 15:**
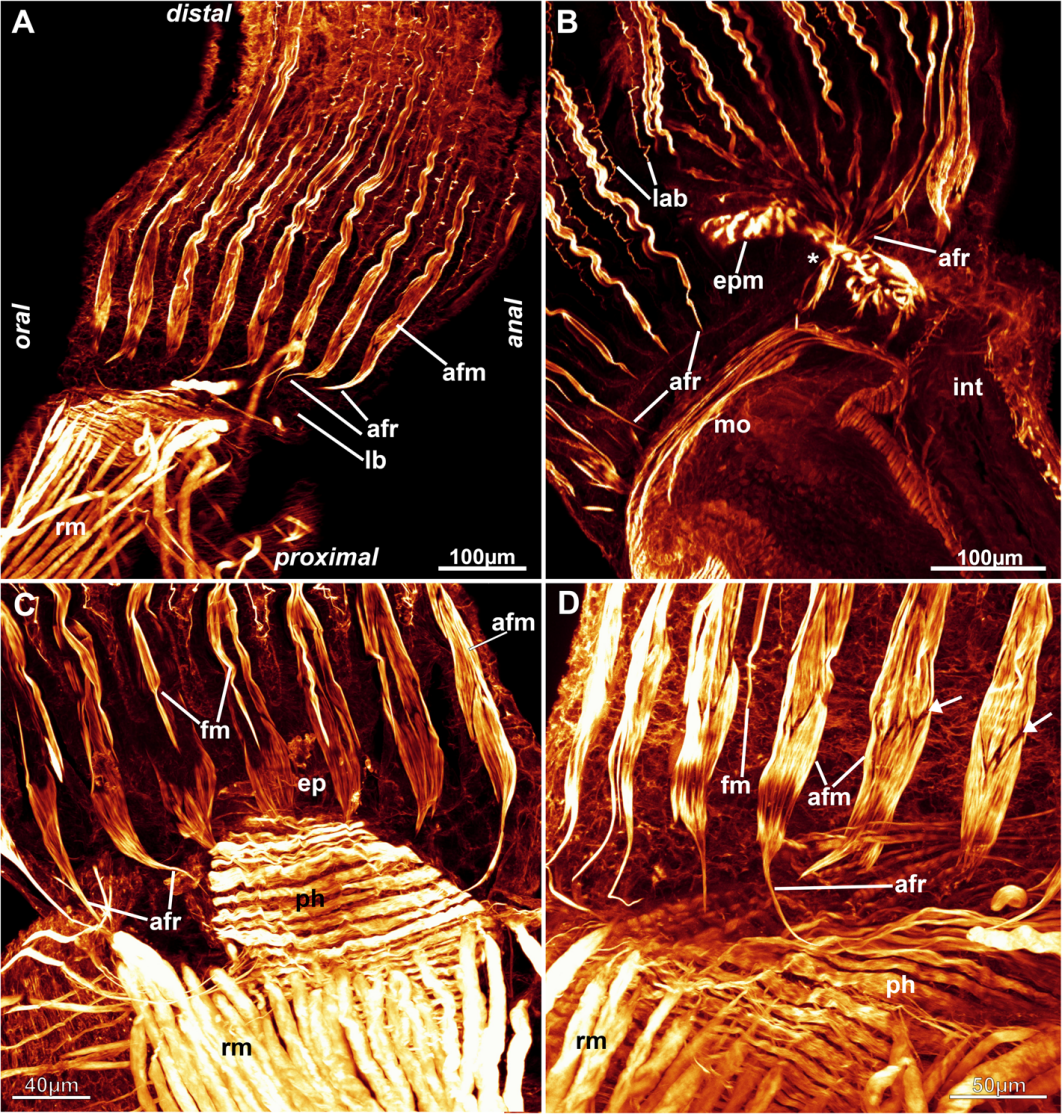
Muscular system of the lophophore of *Stephanella hina*. Confocal laser scanning microscopy stacks based on f-actin labelling. Volume renderings, projections or optical slices. F-actin in glow LUT. **a** Lateral view of a retracted polypide showing the foregut and retractor muscle. **b** Lateral view of the abfrontal muscle roots in the inner lophophoral concavity where the roots merge lateral to the epistome (asterisk). **c** Close up from the anal side showing tentacle muscles. **d** Details of the proximal abfrontal muscle roots. Note the zig-zag arrangement of the abfrontal muscle bands (arrows). Abbreviations: afm – abfrontal muscle band, afr – abfrontal muscle root, ep – epistome, epm – epistome musculature, fm – frontal muscle, int – intestine, lab – lateral actin-rich borders of tentacles, lb. –lophophore base, mo – mouth opening, ph – pharynx, rm. – retractor muscle

Six principal sets of muscles can be distinguished in bryozoans [17, 18] that in *Stephanella hina* consist of 1) body wall muscles in a regular, orthogonal grid of longitudinal and circular muscles (Figs. 4c, e; 13b; 14a, e). The inner longitudinal muscles are, in most parts of the body wall, thicker and more prominent than the circular ones. 2) Apertural muscles consisting of radially arranged peritoneal duplicature bands supplied with longitudinal muscles and separate vestibular dilatators. Both originate from the tentacle sheath close to the vestibular wall and extend towards the lateral body wall. The duplicature bands originate more proximally, directly from the muscles of the tentacle sheath, and the dilatators, more distally from the area of the diaphragmatic sphincter (Figs. 3a, c, d, f; 4a, d). The diaphragmatic sphincter separating the tentacle sheath from the vestibular wall is not distinguishable from the remaining circular vestibular wall musculature. 3) Tentacle sheath muscles that predominantly show thick longitudinal but also thinner circular fibres (Fig. 4 D; 14a, c). The arrangement is reminiscent of that of the body wall, with prominent longitudinal fibres and more delicate circular fibres. 4) Digestive tract muscles present only in a circular arrangement in the lining of the digestive tract (Fig. 11). These are densely arranged into thick muscle bundles over the entire range of the gut. The fibres of the foregut (pharynx and esophagus) and first part of the midgut, the cardia, have striated muscle fibres (although sometimes the striation is not readily evident). The remaining gut shows smooth muscle fibres. Funicular musculature was not detected. 5) Lophophoral muscles. Each tentacle has two longitudinal muscle bands, one on the frontal (i.e., facing the mouth opening) side and one on the abfrontal (i.e., facing the outer edge of the lophophore) side (Fig. 15). The abfrontal muscle begins as a thin thread at the lophophoral base and continues into a broader stack of several muscular areas arranged in a zig-zag manner (Fig. 15d). The remaining abfrontal muscle band extends to the distal tip of the tentacle. In the inner lophophoral concavity, the abfrontal roots converge lateral to the epistome to a single site of origin (Fig. 6d). The frontal muscle band has its roots more distal to that of the abfrontal band in the lophophoral base but does not show any specific rooting in the latter (Fig. 15c, d). It is thinner than the abfrontal muscle and extends along each tentacle as well. Laterally, each tentacle shows a distinct f-actin–rich border (Fig. 15b). With the exception of the epistome, no additional musculature is present on the lophophoral base. The epistome has a series of thick, smooth muscle bundles traversing the epistomial cavity (Figs. 6a, c–e; 15b). In addition, two longitudinal muscle bands are embedded in the lateral lining of the epistomial canal to the anal side of the cerebral ganglion that connects the epistomial coelom with the remaining body cavity (Fig. 6a, b). Several thinner muscle bands appear to run in parallel on the proximo-lateral sides of the ganglion. 6) Retractor muscles are the most prominent and thickest muscles in the zooid. They consist of a series of bundles emerging from a single origin on the body wall that extend to the oral side of the polypide and attach at the lophophoral base, the foregut and parts of the midgut (cardia, caecum) (Fig. 11c). These are exclusively smooth muscle fibres.

### Nervous system

**Fig. 16:**
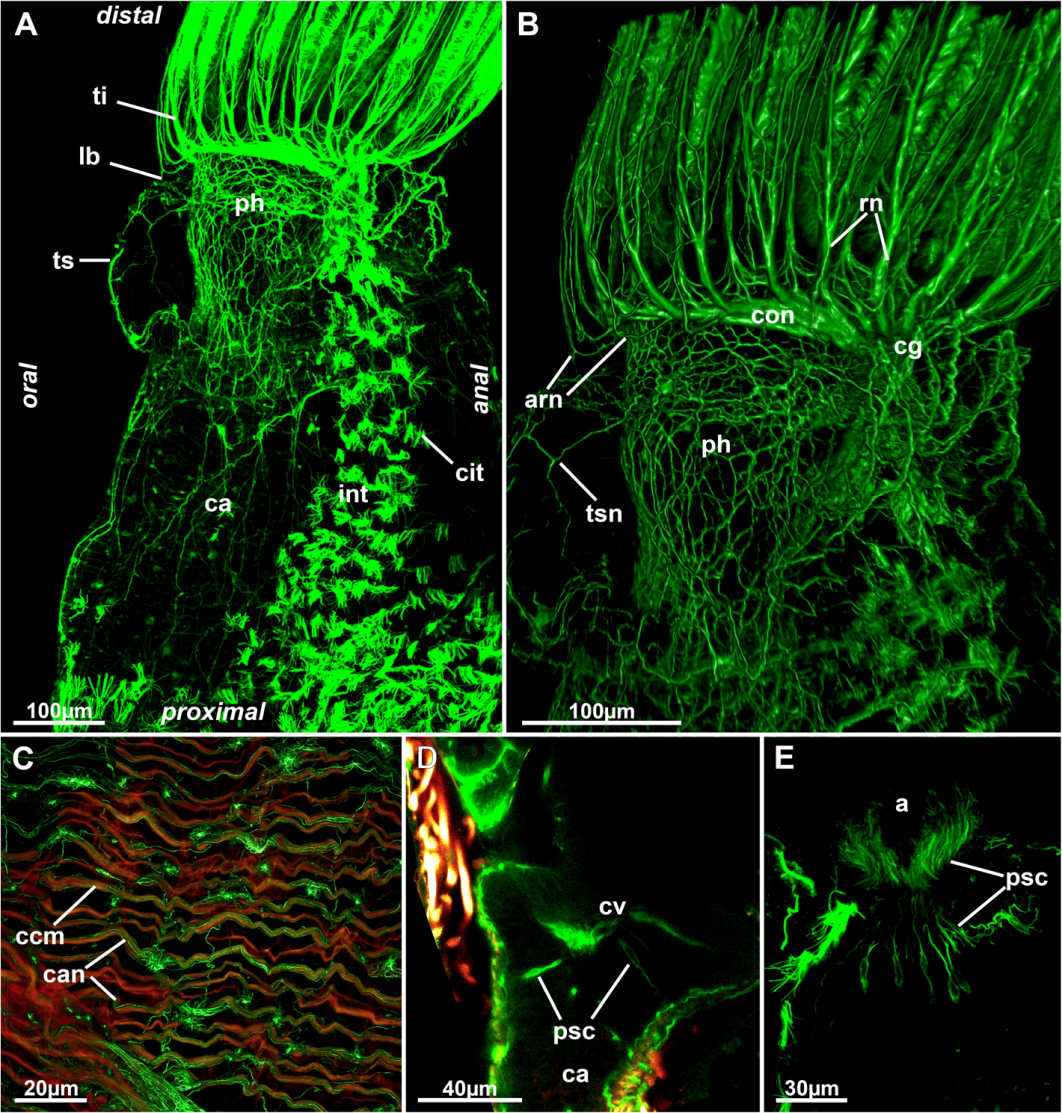
Nervous system of the digestive system of *Stephanella hina*. Confocal laser scanning microscopy stacks based on acetylated alpha-tubulin labelling. Volume renderings, projection or optical slices. Acetylated alpha-tubulin in green LUT, f-actin in glow LUT. **a** General overview of an extended polypide showing dense innervation of the foregut. Note also the dense ciliary bundles on the anal side of the intestine. **b** Details of the foregut shown in A. **c** Innervation of the caecum. **d** Optical section of the foregut-cardia transition showing few presumptive sensory cells projecting into the digestive epithelium. **e** Presumptive sensory cells in the lining of the intestinal wall close to the anus. Abbreviations: a – anus, arn – additional radial nerve, ca – cardia, can – caecum innervation, ccm – caecal muscles, cg – cerebral ganglion, cit – ciliary tufts, con – circum-oral nerve ring, cv – cardiac valve, int – intestine, lb. – lophophoral base, ph – pharynx, psc – presumed sensory cells, rn – radial nerve, ti – tentacle innervation, ts – tentacle sheath, tsn – tentacle sheath innervation

**Fig. 17:**
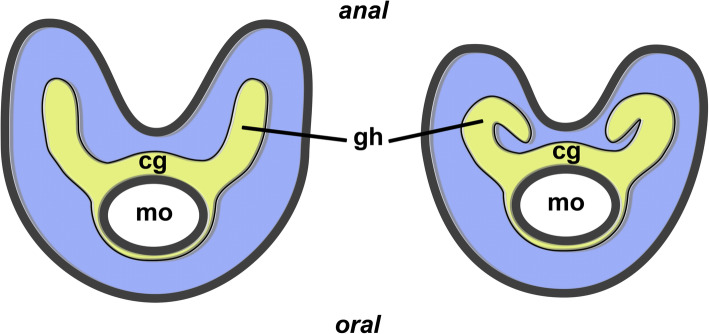
Schematic drawing of the neuronal structures of the lophophoral base of most phylactolaemates (left) and *Stephanella hina* (right). Abbreviations; cg - cerebral ganglion, gh - ganglionic horn, mo - mouth opening

The centre of the nervous system is the cerebral ganglion located at the lophophoral base (Figs. 5; 6f; 7d; 16b). It is adjacent to the anal side of the pharyngeal wall of the gut. The ganglion contains a small cavity (Fig. 7d) that is shifted towards the pharyngeal wall, whereas the neuronal tissue is concentrated on the anal side in the form of a croissant. Lateral to the ganglion, two ganglionic horns or extensions emerge that protrude distally towards the short lophophoral arms. These are slightly curved, bending medially into the direction of the inner arc of tentacles into the lophophoral concavity towards the epistome (Figs. 5; 17).

**Fig. 18:**
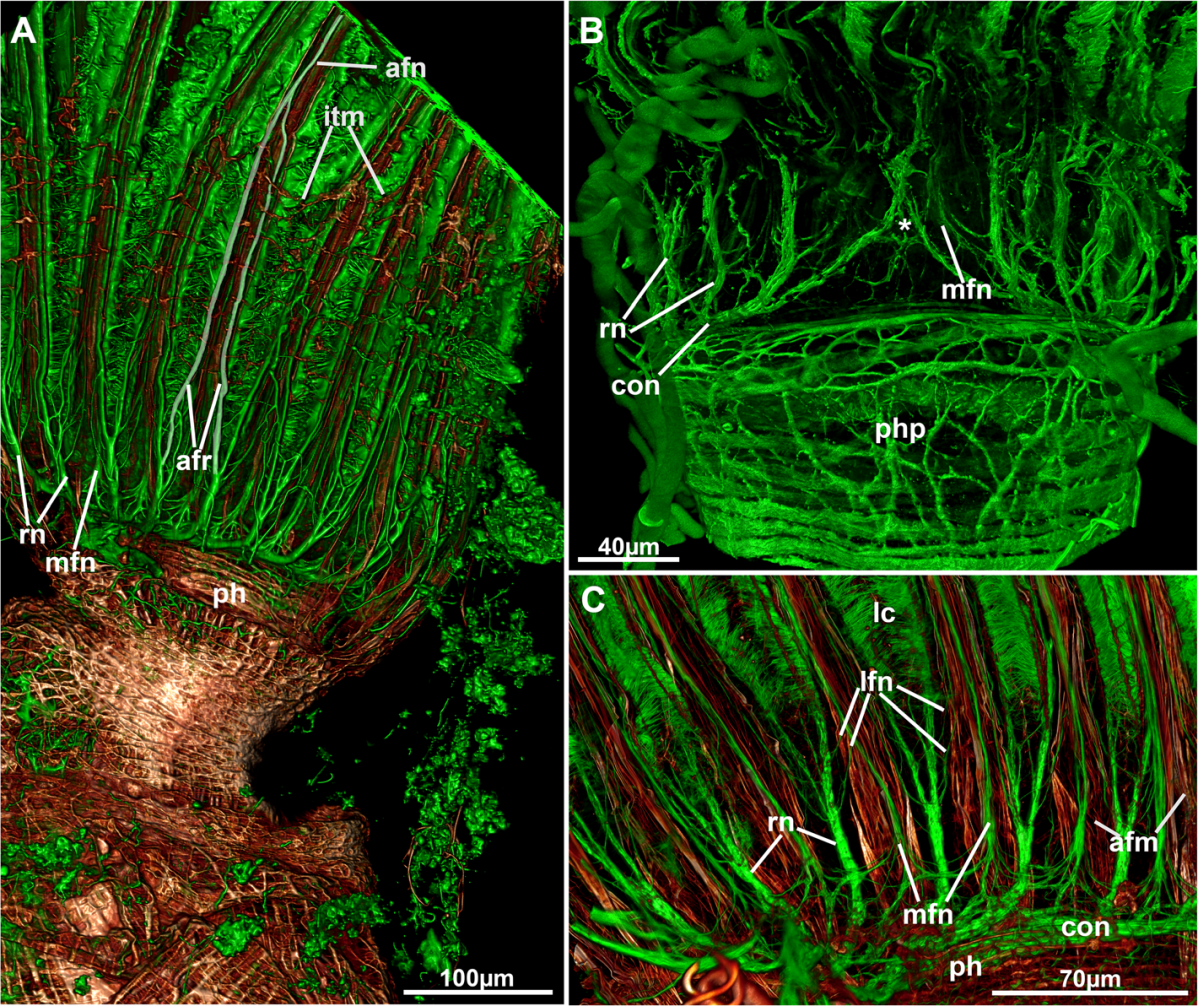
Overview of the lophophoral nervous system of *Stephanella hina*. Confocal laser scanning microscopy stacks based on acetylated alpha-tubulin labelling. Volume renderings. Acetylated alpha-tubulin in green LUT, f-actin in glow LUT. **a** Volume rendering showing an overview of the lophophoral nervous system. One of the abfrontal neurite bundles and its roots have been highlighted in white. **b** Oral view of the circum-oral nerve ring closing medially on the oral side (asterisk). **c** Close-up of the lophophoral base showing radial nerves and their position with respect to the abfrontal tentacle muscle bands. Abbreviations: afm – abfrontal muscle band, afn – abfrontal neurite bundle, afr – abfrontal neurite bundle root, con – circum-oral nerve ring, itm – distal border of the intertentacular membrane, lc – lateral cilia, lfn - latero-frontal neurites, mfn – medio-frontal neurite bundle, ph – pharynx, php – pharyngeal plexus, rn – radial nerve

**Fig. 19:**
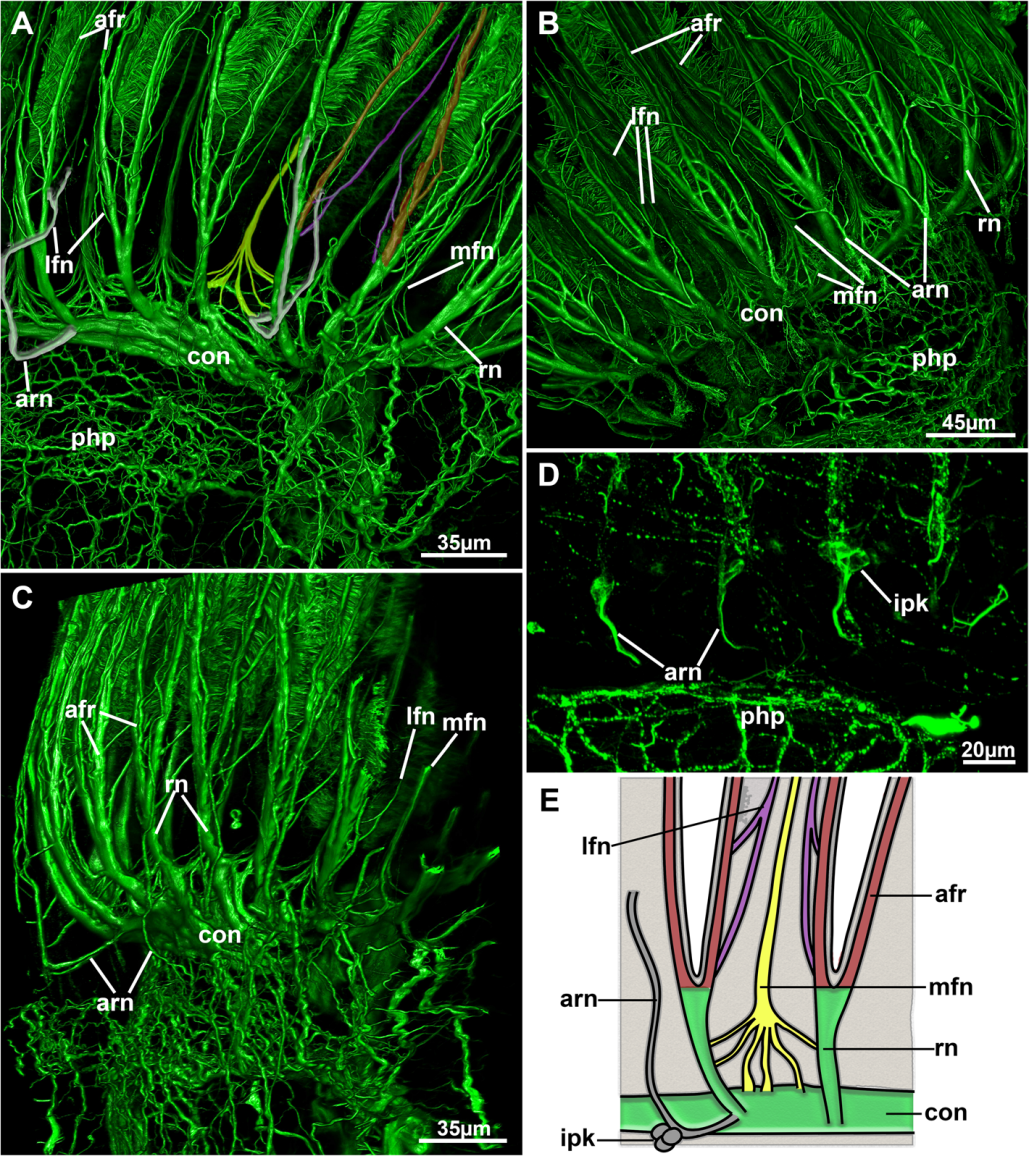
Detail of the tentacle innervation of *Stephanella hina*. **a** Lateral view of the lophophoral base showing the main tentacle neurite branches. One medio-frontal neurite bundle has been marked in yellow, one latero-frontal pair in purple, one pair of abfrontal neurite bundle roots in red and two additional radial neurite bundles in grey. **b** Similar view as in A showing major neurite bundle origins from the circum-oral nerve ring. **c** Lateral view of the lophophoral base showing the traversal of the additional radial nerve/neurite bundle supplying the outer lophophoral base. **d** & **e** Intertentacular perikarya at the additional radial neurite bundle. **d** Optical section. **e** Schematic drawing of the main tentacle neurite bundles. Abbreviations: afr – abfrontal neurite bundle roots, arn – additional radial nerve/neurite bundle, con – circum-oral nerve ring, ipk – intertentacular perikarya, ph – pharynx, lfn – latero-frontal neurite bundles, mfn – medio-frontal neurite bundle, php – pharyngeal plexus, rn – radial nerve

A circum-oral nerve ring (CON) emanates from both lateral sides of the ganglion and encircles the mouth opening. From the ganglion and the CON, intertentacular neurite bundles, the radial nerves, emerge from where most of the tentacle neurite bundles branch off (Figs. 5; 16b; 18; 19a–c). Additional medio-frontal neurite bundles branch from paired roots that emerge either directly from the CON or from the proximal areas of the radial nerves (Figs. 18; 19a, b). In total, there are four distinct tentacle neurite bundles: The medio-frontal neurite bundles emerge most proximally with several roots that merge in the median plane of each tentacle to form a single neurite bundle for each tentacle (Fig. 19a). The roots commonly show several lateral interconnections between the tentacles. The remaining tentacle neurite bundles arise from the prominent intertentacular radial nerves. First, at least two thin roots, one more proximal and another more distal, branch of the radial nerve and traverse frontally to form a latero-frontal neurite bundle on each side of a tentacle, next to the medio-frontal bundle (Figs. 18c; 19a–c). More distally, the radial nerve branches into two thicker bundles that extend to the proximo-lateral and abfrontal sides of each tentacle (Figs. 18a; 19a–c). On each tentacle, these paired latero-abfrontal neurite bundle roots merge further distally into a single abfrontal neurite bundle (Fig. 18a).

Additional radial neurite bundles are present at the lophophoral base. These branch off the root of the radial nerve and extend more proximally towards the outer margin of the lophophoral base (Fig. 19). At the outer margin of the lophophoral base, these bundles frequently lead to a series of two to three perikarya in the epidermal layer of the outer lophophoral base (Fig. 19d, e). Turning distally, the remaining neurite bundles of the additional radial nerve innervate the outer lophophoral base (Fig. 19a–c).

Some samples show distinct neurite bundles extending from the cerebral ganglion into the epistome. These bundles branch directly from the cerebral ganglion oro-distally into the epitome as a fine plexus (Fig. 6f).

In addition to lophophoral innervation, additional neurite bundles extend from the cerebral ganglion and the CON to the visceral parts and the tentacle sheath. The digestive tract shows a diffuse nerve plexus that is most concentrated in the foregut until the cardiac valve (Figs. 16a, b; 18b; 19). There are no distinct concentrated neurite bundles present in the foregut. Innervation of the midgut is rather sparse, with only a few neurite bundles associated with the gut wall. In the caecum, these are associated with the circular muscle bundles (Fig. 16c). No distinct innervation of the intestine could be found. Presumptive sensory cells have been detected in the foregut, cardia and anal areas, emerging from the nerve plexus wedged into the remaining cells of the gut epithelium (Fig. 16d, e).

The tentacle sheath shows a diffuse nerve plexus without any particular concentrated neurite bundles, similar to the visceral innervation. The plexus extends over the entire tentacle sheath (Figs. 14b; 16a, b). Close to the vestibular area, neurite bundles extend via the duplicature bands into the body wall plexus (Figs. 4a, b; 14d).

## Discussion

The general morphology of *Stephanella* is similar to other phylactolaemate species (see [4]); nevertheless, several fine details, particularly of the lophophore structure, are strikingly different from those of any other species.

### Epidermis and vestibular wall

The epidermal structure has seldom been used for comparative analyses. Instead, the glandular morphology of the typical gelatinous families lophopodids, cristatellids and pectinatellids was previously recognized as important for its systematic implications [19]. In addition to specific glandular patches, the so-called white spots, that are restricted to a small number of species [20], two different gland types are generally distinguished: the vacuolar type and the alveolar type. The former are epithelial cells containing a single large vacuole, whereas the latter have numerous smaller vesicles throughout the cytoplasm. Vacuolar cells are also present in *Stephanella hina*, whereas distinct alveolar cells could not be identified in the current study. In addition to large vacuolar inclusions, smaller translucent vesicular structures were found in *S. hina*, but these appeared to also occur within vacuolar gland cells. In general, the high heterogeneity of the endocyst structure in *S. hina* remains poorly understood. It is possible that the presence and thickness of the ectocyst tube is reflected in the glandular composition of the epidermis, especially since the ectocyst is, for the most part, not connected to the epidermis and can also be removed without greater harm to the zooid [14]. Since the ectocyst structure is a diagnostic character for genus classification in plumatellids, the largest taxon of Phylactolaemata, the epidermal structure appears promising for future comparative analyses over the entire range of families and within genera of a specific family, especially in regard to ultrastructure, which was analysed in only a few studies [21, 22] and has never been the specific focus of any.

Likewise, the vestibular wall structure also remains poorly studied (see [4, 19]). It is always a prominent, thickened epithelium as encountered in *S. hina*. With the exception of *Cristatella mucedo*, the integration of glandular cells into the vestibular wall has not been described [19]. *Stephanella hina* shows characteristic, large vacuolar cells (this study), which show little similarity to the glandular cells of *C. mucedo*. In addition, positive anti-acetylated alpha tubulin staining has never been described in this structure for any other phylactolaemate (see ICC studies on phylactolaemates, e.g., [20, 23–25]). The encountered connection with neurite bundles indicates that these cells might be neurosecretory in *S. hina*. In summary, these appear unique, if not autapomorphic, to *S. hina*.

### Lophophore

Phylactolaemates typically possess a horseshoe-shaped lophophore with an epistome at its base protruding over the mouth opening from the anal side [17, 26]. The coelomic system of the phylactolaemate lophophore shows three distinct canals: a median epistomial canal, which extends from the inner peritoneal lining of the gut shanks into the epistome, the ring canal, which is a short canal supplying the oral tentacles of the lophophore, and the forked canal, which interconnects the cavities of the innermost tentacle row in the lophophoral concavity. Unique to *Stephanella hina* is the lack of a distinct, continuous forked canal. In all other phylactolaemates, the latter commences on the lateral, proximal sides of the cerebral ganglion as ciliated openings that lead into two ducts that medially fuse in the distal direction. This medial fusion of the ciliated ducts arches directly above the epistomial coelomic extension located above the cerebral ganglion [17, 22, 27, 28]. The few tentacles in the inner lophophoral concavity are supplied by the forked canal. The general layout in *S. hina* is similar to ciliary patches at the openings of the forked canal ducts, but in *S. hina,* the coelomic cavities do not fuse at the terminal or median ends.

### Epistome

The epistome structure was most recently studied in several phylactolaemate species [17, 21, 22] and always consists of a highly prismatic epithelium of mostly ciliated cells. To date, distinct holocrine secretion via the epistome epithelium (or the underlying gut epithelium surrounding the mouth opening), as encountered in the current study on *S. hina,* has not been reported in any other species. Interestingly, the distribution of these secretory processes correlates with the reddish coloration of the same areas in live animals [14]. Colorations or tinges of the epistome are common in phylactolaemates, e.g., with *Cristatella mucedo* having a brownish hue (and *Pectinatella magnifica* a bright red coloration in the epistome, e.g., [3, 4]). In such prominent forms with very large zooidal sizes, these colorations are very evident. However, distinct darker hues are also present in other phylactolaemates, such as plumatellids.

Excretion is a little studied process in bryozoans and often seems to be associated with coelomocytes [2]. The secretory mechanism found in *Stephanella hina* in the present study seems to be newly discovered but poorly understood. The high variability of the epistome structure observed in this study indicates that this process is probably influenced by external factors such as (e.g., physical or temperature-induced) stress. Likewise, the coelomocyte abundance in *S. hina* appears to be stress-related [Schwaha, personal observation].

### Digestive tract

The digestive tract of *S. hina* is similar to that of other phylactolaemates [2, 17]. A persistent problem is the terminology used for and homology of the foregut. In the last summary on bryozoan digestive systems, the phylactolaemate pharynx was always considered the first area of the foregut that bears cilia [29]. Consequently, only the short area surrounding the mouth opening should be addressed as the ‘pharynx’ in *S. hina* and the remaining, particularly vacuolated epithelium as the esophagus until the cardiac valve. However, several studies (including the present study) frequently label the upper vacuolated area the pharynx (see [21]). This mostly results from the terminology applied to myolaemates, which are characterized by a myoepithelial, suction pharynx also characterized by vacuolated cells [2, 29]. Similar to *S. hina*, only a short area close to the mouth opening carries cilia, whereas most of the vacuolated area lacks them. Both, however, are myoepithelial and show distinct cross-striated muscle filaments in their lateral linings. Beyond the vacuolated cells, the esophagus of myolaemates is not myoepithelial or vacuolated. Hence, the mixture of different cytological characteristics makes it difficult to truly assign the parts of the gut.

The typical ciliated pharyngeal area of other phylactolaemates is frequently longer than in *S. hina* [29], whereas the vacuolated area of the foregut has either been described with basally located nuclei and single large vacuoles [17] or, as in *S. hina,* with nuclei in the middle of the cells with vacuoles on the base and distal areas [21]. As shown in the present study, the nuclei are very small and appear not very active, suggesting that the foregut is mere a mechanical transport tube (see also [29]). In the plumatellid *Hyalinella punctata,* presumptive sensory cells are also embedded into this area of the gut [25], but these were not very abundant in *S. hina*. The remaining gut of *S. hina* shows no distinct differences from any other phylactolaemate.

Additional studies should comparatively analyse all six major phylactolaemate families regarding the anatomy and cytological specifications of the foregut. This should aid in redefining the terminology in terms of more criteria than just ciliation and also investigate whether morphological details of the gut might have any systematic or phylogenetic value.

### Myoanatomy

The myoanatomy of phylactolaemates has been studied recently with f-actin staining in four of its six major families, including Plumatellidae and Fredericellidae by Schwaha & Wanninger [30] and Pectinatellidae and Cristatellidae by Gawin et al. [31]. The current investigation on the fifth family, Stephanellidae, with its current sole representative *Stephanella hina*, shows that the main muscular systems are very similar to previous descriptions. Concerning the six abovementioned muscular systems, the following can be summarized. 1) The body wall possesses a regular muscular grid as present in other phylactolaemates [2, 4]. A diagonal muscle layer, as found in *Pectinatella* or lophopodids [31], was not found. Hence, a regular orthogonal grid is most likely the ancestral condition. 2) The apertural muscles with duplicature bands and vestibular dilatators are in accordance with most previous descriptions of phylactolaemates [15, 17]. Accordingly, since the duplicature bands attach to the distal tentacle sheath in *S. hina*, the situation is similar to that of most phylactolaemates except lophopodids where the bands attach at the diaphragmatic sphincter [15]. Hence, the more ubiquitous insertion of duplicature bands at the tentacle sheath rather than the diaphragm appears to be the ancestral condition for phylactolaemates. This is also supported in non-phylactolaemates where the bands always insert at the tentacle sheath [2]. The insertion area at the diaphragm in reported lophopodids is probably a derived and apomorphic feature. Most bryozoans, including many phylactolaemates, have a pronounced vestibular wall area, which, however, is little pronounced in *S. hina*. Hence, the differentiation of the diaphragmatic sphincter structures is not as evident as in other species (see [30, 31]).

3) The tentacle sheath muscles comprise more prominent longitudinal and circular muscles. The arrangement thus reflects the general arrangement of the body wall musculature, as also seen in *S. hina*. This supports the notion that a regular grid of orthogonal musculature is the ancestral condition for phylactolaemates (see [17, 30, 31]). The condition of plumatellids solely bearing longitudinal muscles in the tentacle sheath is thus probably derived, similar to the remaining bryozoans, which also usually only possess longitudinal muscle fibres in the tentacle sheath [2, 17]. 4) The digestive tract is similar to that in other described phylactolaemates (see [17, 30]) in that it is supported only by circular muscles. No longitudinal muscle fibres were found, as indicated for the lophopodid *Asajirella gelatinosa* [21]. In contrast to other species, *S. hina* has smooth muscle fibres in the caecum lining, which is striated in other forms (see above citations). In addition, the arrangement of the muscle bands is very dense in other phylactolaemates, whereas *S. hina* shows a rather loose pattern, particularly in the caecum. In contrast to all other analysed phylactolaemates, *S. hina* does not show longitudinal muscles in the funiculus (see [2]).

5) In all bryozoans, including phylactolaemates, the tentacles are supplied with two longitudinal muscle bands [17]. These are mostly smooth fibres, which is also the case in *Stephanella hina*. The lophophoral base area where these muscle bands insert differs between phylactolaemates and myolaemates, with a series of small muscular elements being present in the latter. In S*. hina*, these rootlets are negligible since the frontal muscle bands appear more distally and show no rooting of any sort, and the abfrontal muscles only have a thin elongated proximal extension. This differs from the previous description of the other four families, where the frontal tentacle muscles show one to two different rootlets, which on the oral side are even connected to the pharyngeal musculature. Likewise, the abfrontal muscle bands have a characteristic arrangement not found in *S. hina*, where only a series of a few stacked muscle bundles, intertwined in a zig-zag fashion, are present. Consequently, *S. hina* entirely differs in its basal root structure of the tentacle musculature from the other four analysed families [30, 31].

The epistomial musculature can show two different patterns: muscle fibres embedded in the epithelial linings or individual muscle fibres traversing the epistomial coelom/cavity(see [17, 28, 30, 31]), although a mixture of both systems was also detected in the plumatellid *Hyalinella punctata* [31]. In *S. hina,* the second state, involving individual fibres, is present and shared by lophopodids and pectinatellids, whereas the three remaining families, Cristatellidae, Fredericellidae and Plumatellidae, share the first configuration. It thus remains difficult to assess which type of epistome musculature is ancestral, but since lophopodids and stephanellids are early branching, it seems reasonable to assume individual traversing fibres as the ancestral condition.

Muscles associated with the proximal border of the lophophoral ring canal were detected in *Cristatella mucedo*, *Pectinatella magnifica* and *Hyalinella punctata* [31] and were probably overlooked in other plumatellids and fredericellids (Schwaha, personal observation). We could not detect any muscles associated with the ring canal, which indicates that these evolved within phylactolaemates or were lost by *Stephanella*.

6) The retractors show no difference from those of any studied phylactolaemates (see references above). They consist of numerous bundles originating from the body wall and attach at several locations on the oral side of the polypide. They are of a smooth fibre type.

### Nervous system

The general structure of the nervous system is similar to that of other bryozoans [17, 23, 25, 32]. The most striking difference is the unique arrangement of the ganglionic horns in *Stephanella hina*. These proceed distally from the lateral sides of the cerebral ganglion and bend medially to extend towards the lateral margins of the epistome. In other phylactolaemates, the ganglionic horns extend in a straight line distally towards the tip of the lophophoral arms [23, 27, 31, 33], and see Fig. 17). Since *S. hina* is the only representative with such an unusual condition, it is concluded that this characteristic (like the general lophophore situation, see above) is apomorphic. The traverse of the bent ganglionic horns is reminiscent of the epistomial nerve (see [34]), but such a nerve could not be detected in more recent analyses. Instead, the epistome is innervated by a plexus emanating directly from the cerebral ganglion, as also described for *Hyalinella punctata* [25]:

Additional differences in the nervous system are evident in the detailed tentacle innervation that in *Stephanella hina* appears to completely lack latero-abfrontal neurite bundles in each tentacle, which are otherwise found in all other phylactolaemates [23, 25] and most other lophophorates [5]. The abfrontal neurite bundle is always formed by the medial fusion of two intertentacular roots. In other phylactolaemates, the lateral roots continue as latero-abfrontal neurite bundles. However, *S. hina* seems to be the first phylactolaemate recently investigated that lacks any sign of these latero-abfrontal neurite bundles. The current data do not support any abfrontal lateral neurite bundles, but to completely verify the presence or absence of these thin neurites, transmission electron microscopy would be necessary.

In addition to the apparent lack of latero-abfrontal neurite bundles, the roots of the medio-frontal bundles mostly emerge directly from the circum-oral nerve ring (CON), with only a few emerging from the radial nerves. The latter condition is described for most other phylactolaemates [23, 25]. Hence, the condition in *S. hina* is reminiscent of the medio-frontal nerves of myolaemate bryozoans that show a similar origin with mostly two roots emerging intertentacularly from the CON [16, 17, 35, 36].

Concerning the remaining, more peripheral neuronal elements, the plexus innervating the digestive tract and tentacle sheath is more diffuse and does not show more prominent neurite bundles on either the oral or anal side of the polypide, which was reported for all other species [23, 25, 32, 37]. Concentrated neurite bundles, as found in most phylactolaemates, are also the usual condition in myolaemates that lack a plexus in these areas. The remaining gut shows as little innervation with staining against acetylated alpha-tubulin as previously reported for *Hyalinella punctata* [25] and *Cristatella mucedo* [38]. Distinct intraepithelial, probably sensory, cells, as found in *S. hina,* were also encountered in the aforementioned species.

## Conclusions

Although *Stephanella hina* is clearly a phylactolaemate and possesses many morphological characteristics similar if not identical to those of other members of the clade, there are numerous unique characteristics underlining the special position of this little studied species. These are most apparent in the structure of the lophophore, including the arrangement of the forked canals in the inner lophophoral arc and the ganglionic horns. Additional finer details, such as the lack of funicular musculature, latero-abfrontal neurite bundles or smooth caecum musculature, support that *S. hina* is a distinct family among Phylactolaemata (see also [14]). Although often regarded as one of the earliest branches, if not a sister group, to the remaining phylactolaemate families, it remains difficult to assess whether these unique characteristics are plesiomorphic or apomorphic.

## Data Availability

Data are available upon reasonable request.
